# ID3 promotes homologous recombination via non-transcriptional and transcriptional mechanisms and its loss confers sensitivity to PARP inhibition

**DOI:** 10.1093/nar/gkab964

**Published:** 2021-10-28

**Authors:** Ali Bakr, Joschka Hey, Gianluca Sigismondo, Chun-Shan Liu, Ahmed Sadik, Ashish Goyal, Alice Cross, Ramya Lakshmana Iyer, Patrick Müller, Max Trauernicht, Kersten Breuer, Pavlo Lutsik, Christiane A Opitz, Jeroen Krijgsveld, Dieter Weichenhan, Christoph Plass, Odilia Popanda, Peter Schmezer

**Affiliations:** Division of Cancer Epigenomics, German Cancer Research Center (DKFZ), INF280, 69120 Heidelberg, Germany; Division of Cancer Epigenomics, German Cancer Research Center (DKFZ), INF280, 69120 Heidelberg, Germany; Heidelberg University, Faculty of Biosciences, 69120 Heidelberg, Germany; Division of Proteomics of Stem Cells and Cancer, German Cancer Research Center (DKFZ), INF581, 69120 Heidelberg, Germany; Division of Cancer Epigenomics, German Cancer Research Center (DKFZ), INF280, 69120 Heidelberg, Germany; DKTK Brain Cancer Metabolism Group, German Cancer Research Center (DKFZ), 69120 Heidelberg, Germany; Division of Cancer Epigenomics, German Cancer Research Center (DKFZ), INF280, 69120 Heidelberg, Germany; Division of Cancer Epigenomics, German Cancer Research Center (DKFZ), INF280, 69120 Heidelberg, Germany; Imperial College London, London, SW7 2AZ, UK; Division of Cancer Epigenomics, German Cancer Research Center (DKFZ), INF280, 69120 Heidelberg, Germany; Division of Cancer Epigenomics, German Cancer Research Center (DKFZ), INF280, 69120 Heidelberg, Germany; Division of Cancer Epigenomics, German Cancer Research Center (DKFZ), INF280, 69120 Heidelberg, Germany; Division of Cancer Epigenomics, German Cancer Research Center (DKFZ), INF280, 69120 Heidelberg, Germany; Division of Cancer Epigenomics, German Cancer Research Center (DKFZ), INF280, 69120 Heidelberg, Germany; DKTK Brain Cancer Metabolism Group, German Cancer Research Center (DKFZ), 69120 Heidelberg, Germany; Neurology Clinic and National Center for Tumor Diseases, Heidelberg University Hospital, 69120 Heidelberg, Germany; Division of Proteomics of Stem Cells and Cancer, German Cancer Research Center (DKFZ), INF581, 69120 Heidelberg, Germany; Heidelberg University, Medical Faculty, INF672, 69120, Heidelberg, Germany; Division of Cancer Epigenomics, German Cancer Research Center (DKFZ), INF280, 69120 Heidelberg, Germany; Division of Cancer Epigenomics, German Cancer Research Center (DKFZ), INF280, 69120 Heidelberg, Germany; German Cancer Consortium (DKTK), INF280, 69120 Heidelberg, Germany; Division of Cancer Epigenomics, German Cancer Research Center (DKFZ), INF280, 69120 Heidelberg, Germany; Division of Cancer Epigenomics, German Cancer Research Center (DKFZ), INF280, 69120 Heidelberg, Germany

## Abstract

The inhibitor of DNA-binding 3 (ID3) is a transcriptional regulator that limits interaction of basic helix-loop-helix transcription factors with their target DNA sequences. We previously reported that ID3 loss is associated with mutational signatures linked to DNA repair defects. Here we demonstrate that ID3 exhibits a dual role to promote DNA double-strand break (DSB) repair, particularly homologous recombination (HR). ID3 interacts with the MRN complex and RECQL helicase to activate DSB repair and it facilitates RAD51 loading and downstream steps of HR. In addition, ID3 promotes the expression of HR genes in response to ionizing radiation by regulating both chromatin accessibility and activity of the transcription factor E2F1. Consistently, analyses of TCGA cancer patient data demonstrate that low ID3 expression is associated with impaired HR. The loss of ID3 leads to sensitivity of tumor cells to PARP inhibition, offering new therapeutic opportunities in ID3-deficient tumors.

## Graphical Abstract

**Graphical Abstract:**
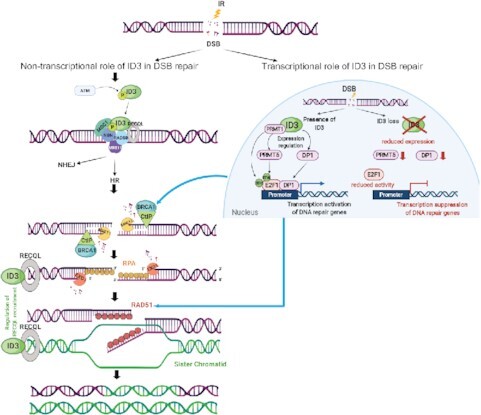
Dual role of ID3 in promoting homologous recombination.

## INTRODUCTION

Genomic stability is continuously challenged by different types of DNA lesions which arise in each cell throughout its lifetime. DNA lesions result from either endogenous genotoxic insults such as by-products of cellular metabolism and inaccurate DNA replication or from exogenous exposures to DNA damaging agents such as ionizing radiation (IR) or cytotoxic chemicals. Among many types of DNA lesions, DNA double-strand breaks (DSBs) are considered the most harmful because unrepaired or incorrectly repaired DSBs can lead to oncogenic chromosomal aberrations, such as deletions and translocations. Those aberrations are associated with developmental defects, immunodeficiency, neurodegenerative disorders, sterility, radiosensitivity and cancer development (1–3). In order to ensure that cells pass accurate copies of their genome on to the next generation, a cellular DNA damage response (DDR) is activated upon DSB induction. This response includes the activation of specific kinases such as ATM, ATR and DNA-PKcs, which subsequently phosphorylate several downstream substrates to initiate molecular DDR events involving cell cycle arrest, transcriptional as well as post-translational regulation of repair-related proteins, and recruitment of these proteins to the site of damage (4–6). Two mechanistically distinct pathways have evolved to eliminate DSBs from the genome non-homologous end joining (NHEJ) and homologous recombination (HR). NHEJ is active almost throughout the cell cycle and ensures that DSB ends are held in proximity to permit their fast direct ligation (7,8). For this reason, NHEJ is considered to be an error-prone repair pathway. In contrast, HR is an error-free mechanism using the sister chromatid as a template. Accordingly, HR is only active during the S and G2 cell cycle phases (9,10).

The inhibitor of DNA-binding 3 (ID3) is a transcriptional regulator protein that acts by forming heterodimers with basic helix-loop-helix (bHLH) transcription factors, thus limiting their binding to DNA (11,12). Alterations of the *ID3* gene such as amplifications, deletions and mutations have been identified in several types of cancers (13). In addition to the known biological function of ID3 as a regulator of transcription and differentiation, it was shown to be involved in cell cycle regulation of normal and pathogenic pancreatic ductal cells (14). Depletion of ID3 or overexpression of its downstream target TCF3 induced cell cycle arrest and reduced cellular proliferation. In contrast, overexpression of ID3 in human pancreatic β-cells was not able to upregulate the proliferation markers Ki67, p-Cyclin E and pH3, but it stimulated the formation of localized BrdU nuclear *foci*, which are associated with DNA damage (15). Further evidence for an association of deregulated ID3 expression with DDR was provided by several studies. O’Brien *et al.* observed that the loss of ID3 resulted in hypersensitivity of colon cancer-initiating cells to oxaliplatin (16). This inter- and intra-strand crosslinking agent causes replication-associated damage which requires the HR repair machinery to be properly repaired. Furthermore, we previously reported that pancreatic acinar cell carcinoma displayed a loss of ID3 protein (17). Such loss was accompanied by high genomic instability and mutational signatures, which are associated with DNA repair defects, particularly in HR. In another study, the results of a yeast two-hybrid screen using the C-terminal fragment of human MDC1 as the bait identified ID3 among the interacting proteins (18). ATM-dependent phosphorylation of ID3 at Ser65 within its HLH domain facilitates the interaction between MDC1 and γH2AX. It is, however, not clear whether MDC1 is the only candidate interacting with ID3 or whether additional repair proteins are involved. Since ID3 is known to be associated with transcriptional regulation, it might also be involved in gene regulation following DNA damage. In order to systematically explore this, we conducted proteomic and transcriptomic analyses following DNA damage induction with IR in wild-type and ID3-depleted cells.

Our results show that ID3 affects DNA repair *via* at least two different mechanisms. First, ID3 can directly interact with DSB repair core proteins such as RAD50, NBS1 and RECQL. Independent of the interaction with MDC1, ID3 can form two distinct complexes, with NBS1 and RAD50 acting on early steps of DSB repair, and more downstream with RECQL to facilitate RAD51 loading. Second, ID3 can induce transcriptional changes of genes involved in the HR and Fanconi Anemia (FA) pathways in response to ionizing radiation. It regulates chromatin accessibility of repair gene promoter regions as well as the activity of the E2F1 transcription factor. Analyzing data sets of The Cancer Genome Atlas (TCGA), we observed direct correlations between ID3 expression and the expression of genes involved in HR-related pathways in several tumor entities. This supports that tumors with low ID3 expression are associated with an impaired HR. Finally, we show that loss of ID3 triggers cellular sensitivity to PARP inhibition, thus offering new therapeutic options for ID3-deficient cancers.

## MATERIALS AND METHODS

### Reagents

Cisplatin (Teva GmbH, Cisplatin Teva^®^). ATM inhibitor KU55933 (Calbiochem, Cat#118500). PARP inhibitor (olaparib) (Selleckchem, Cat# S1060). Protease inhibitor cocktail (Roche Diagnostics, Cat#11836170001). Phosphatase inhibitor cocktail (Roche Diagnostics, Cat#04906837001). Vectashield Antifade mounting medium (Vector Laboratories, Cat#H-1000). bis-Benzamide H33342 trihydrochloride (Sigma, Cat#B2261). Crystal violet (Merck, Cat# C-0775). Puromycin (Merck, Cat# P8833). Geneticin disulfate (G418) (Roth, Cat#2039.3). Penicillin-Streptomycin (Sigma, Cat#P0781). Novex ECL HRP Chemiluminescent Kit (Invitrogen, Cat#WP20005). High sensitivity HRP Chemiluminescent Kit (Merck, Cat#WBKLS0500). PVDF membrane (Thermo Fisher, Cat#88520). Anti-Flag magnetic beads (Thermo Fisher, Cat#A36798). Magna ChIP Protein A magnetic beads (Merck, Cat#16-661). ChIP-Grade Protein G magnetic beads (Cell Signaling, Cat#9006S). Agencourt AMPure XP (Beckman Coulter, Cat#A63880). SuperScript™ III First-Strand Synthesis (Thermofisher, Cat#18080–051). PrimaQUANT™ CYBR green kit (Steinbrenner Laborsysteme, Cat#SL-9902). EndoFree Plasmid Maxi kit (Qiagen, Cat#12362). MinElute PCR Purification kit (Qiagen, Cat#28006). DNeasy kit (Qiagen, Cat#69506). Lipofectamine DharmaFECT 1 (Dharmacoon, Cat# T-2001-03). TransIT-LT1 (Mirus Bio, Cat#MIR 2300). pCBASceI (Addgene, Cat#26477). Q5 high-fidelity DNA polymerase (NEB, Cat#M0491S). Neon™ Transfection System (Thermo Fisher, Cat#MPK10025). Alt-R^®^ CRISPR-Cas9 crRNA (IDT technologies). Alt-R® CRISPR-Cas9 tracrRNA (IDT technologies). Agilent RNA 6000 Nano Kit (Agilent, Cat#5067-1511). Agilent High Sensitivity DNA Kit (Agilent, Cat#5067-4626).

### Antibodies

Rabbit-anti-ID3 (Cell Signaling, Cat#9837) and for IP of endogenous ID3 we used agarose beads conjugated with mouse-anti-ID3(Santa Cruz, Cat#sc-56712). Mouse-anti-beta-Actin (Santa Cruz, Cat#sc-47778). Mouse-anti-Vinculin (Santa Cruz, Cat#sc-25336). Mouse-anti-phospho-Histone H2A.X (S139) (Merck, Cat#05-636). Rabbit-anti-phospho-Histone H2A.X (S139) (Abcam, Cat# ab2893). Rabbit-anti-RAD51 (Calbiochem, Cat#PC130). Rabbit-anti-RAD51 (Abcam, Cat#ab176458). Rabbit-anti-XRCC4 (GenTex, Cat#GTX109632). Rabbit-anti-phospho-RPA (S4/S8) (Bethyl, Cat#A300-245A). Mouse-anti-Histone H2B (Abcam, Cat#ab52484). Rabbit-anti-DNA-PKsc (Cell Signaling, Cat#4602). Rabbit-anti-CtIP (Abcam, Cat#70163). Goat-anti-MDC1 (Santa Cruz, Cat#sc-27737). Rabbit-anti-MDC1 (Abcam, Cat#ab11169). Rabbit-anti-NBS1 (Novus Biologicals, Cat#NB100-143). Mouse-anti-RAD50 (Abcam, Cat#ab89). Rabbit-anti-RECQL (Abcam, Cat#ab151501). Mouse-anti-RPA32 (Santa Cruz, Cat#sc-53496). Mouse-anti-Flag-HRP (Sigma, Cat#A8592). Mouse-anti-IgG (Santa Cruz, Cat#sc-2025). Mouse-anti-CenpF (BD bioscience, Cat#610768). Goat-anti-mouse IgG-HRP (Cell Signaling, Cat#7076P2). Goat-anti-rabbit IgG-HRP (Cell Signaling, Cat#7074S). Goat-anti-mouse IgG-AlexaFluor 594 (Molecular Probes, Cat#A11005). Goat-anti-rabbit IgG-AlexaFluor 488 (Molecular Probes, Cat#A11008).

### Laboratory instruments

FACSCanto™ II Flow Cytometer (BD, Cat#338960). 2100 Bioanalyzer Instrument (Agilent, Cat#G2939BA). 4150 TapeStation System (Agilent, Cat#G2992AA). Amersham Imager 680 (GE Healthcare, Cat#29270769). Axioplan 2 imaging microscope (Zeiss). LightCycler^®^ 480 (Roche, Cat#05015243001). microTUBE AFA Fiber Pre-Slit Snap-Cap (Covaris, Cat#80606). M220 Focused-ultrasonicator (Covaris). Gammacell® 40 Exactor (Theatronics). Thermocycler (Eppendorf).

### Biological resources

MIA PaCa-2 (human pancreatic adenocarcinoma), DU145 (human prostate cancer), U2OS (human osteosarcoma), PSN-1 (human pancreatic adenocarcinoma), LNCap Clone FGC (human prostate cancer) and HEK293T cells were cultured in Dulbecco's modified Eagle's medium (DMEM) supplemented with 10% (vol/vol) fetal bovine serum (BioChrom), 100 U/ml penicillin, 100 μg/ml streptomycin (Sigma-Aldrich). AID-DIvA cells (originally U2OS cells integrated with AsiSI- expressing vector) were cultured as in (19), for maintenance and selection culture medium was additionally supplemented with 800 μg/ml Geneticindisulfate (G418) (Roth). Upon addition of 4-hydroxytamoxifen to the culture medium, the AsiSI enzyme is localized to the nucleus and generates several DSBs in the genome. For the selection of ID3-KO cells stably expressing Flag-ID3 (ID3-rescue) AID-DIvA cells additionally 2 μg/ml puromycin (Merck) was used. U2OS-EJ5 and U2OS-DR were cultured as in ref. (20), for maintenance and selection 2 μg/ml puromycin (Merck) was used. Cells were maintained in a humidified incubator with an atmosphere of 5% CO_2_ at 37°C. All cells were originally obtained from the ATCC cell repository, AID-DIvA cells were kindly provided by Dr. G. Legube (University of Toulouse, France) and U2OS-EJ5 and U2OS-DR were kindly provided by Dr. J. Stark (City of Hope center, USA). Cells were routinely tested to be mycoplasma-free. The Competent Bacterial Strain DH5α E. coli was used for transformations.

### Statistical analyses

Unless stated, GraphPad Prism v5 software was used to create graphs, perform statistical tests, and calculate *P*-values. Statistical analyses for RNA-seq and ATAC-seq were performed using R version 3.6 (21). Figure 7I was created with BioRender.com.

### siRNA and plasmid transfection

Set of 4 siGENOME upgrade siRNAs were obtained from Dharmacoon, pooled together and transfected using Lipofectamine DharmaFECT1 (Dharmacon) according to the manufacturer's protocol. Plasmid transfections were carried out using TransITLT1 (Mirus Bio) according to the manufacturer's protocol. For siRNA and plasmid co-transfections, plasmids were transfected 48h after siRNA treatment. See Supplementary Table S8.

### Generation of ID3-knockout cells

Two separate CRISPR guide RNA sequences (crRNA#1: 5′-CCGGGGCCGAGGGAAGGGCC(CGG)-3′ and crRNA#2: 5′- TGGGGGCCATCAGGGGGTCC(AGG)-3′) targeting the exon1 of ID3 open reading frame were designed using IDT Alt-R® CRISPR-Cas9 guide RNA design tool and were ordered as Alt-R® CRISPR-Cas9 crRNA (IDT technologies). 22 pmol crRNA was annealed with 22 pmol Alt-R^®^ CRISPR-Cas9 tracrRNA (IDT technologies) by heating at 95°C for 5 min and cooling down to room temperature. 18 pmol Alt-R® Cas9 nuclease was added to the annealed crRNA:tracrRNA complex and incubated for 15 min at room temperature (final volume = 1 μl). 200 000 cells were resuspended in 10 μl neon electroporation Buffer R (Thermo Scientific). The Cas9:crRNA:tracrRNA complex was added to the cells along with 1 μl of 25 μM electroporation enhancer (IDT technologies). Cells were electroporated using 10 μl Neon electroporation tips with the following settings: 1050 V, 30 ms, 2 pulses. Cells were transferred to 12-well plates containing 1 ml growth media. Seventy-two hours post-electroporation, single cells were sorted into 96-well plates and allowed to proliferate. Single-cell clones thus obtained were screened for ID3 knockout using immunoblotting and Sanger sequencing.

### Generation of ID3 expressing vectors

The ID3 full length, phospho-mutant ID3-S65E and -S65A, and empty-pLVX vector were cloned into XhoI- BamHI sites of pLVX-Puro to form ID3-pLVX (provided by Biocat). It is a lentiviral plasmid that directs the synthesis of human ID3 with GFP, HA and 3 Flag tags fused in the N-terminal of the ID3 protein. The ID3-pLVX plasmid was co-transfected with packaging plasmid psPAX2 and pMD2.G (both from PlasmidFactory GmbH & Co. KG) into HEK293T cells by TransIT LT1 transfection reagent, and the medium was replaced with fresh medium the next day. The virus-containing supernatant was harvest 2 days post-transfection and subsequently transduced into ID3-KO cells (AID-DIvA cells and U2OS cells). Indicated cell populations were stably established by puromycin resistance and confirmed by western blot analysis.

### Whole-cell protein extracts and western blotting

Whole-cell extracts and western blotting were performed as described in ref. (22). Protein extracts were prepared for SDS-PAGE in Laemmli buffer (10% SDS, 300 mM Tris–HCl, 10 mM b-mercaptoethanol, 50% glycine, 0.02% bromophenol blue). Separated proteins were transferred to a PVDF membrane, blocked at RT for 1 h in 5% skimmed milk in TBS–0.2% Tween and incubated with primary antibodies overnight at 4°C. Membranes were then washed and incubated with HRP-conjugated secondary antibodies at RT for 1 h. Detection was done by the HRP Chemiluminescent Substrate Reagent Kit Novex ECL (Invitrogen) or high sensitivity Kit (Merck Millipore). Measurement is performed using the Amersham imager 680 GE (Healthcare). Quantification of blots was performed using ImageJ. All protein concentrations were determined using a BCA assay (Sigma Aldrich) and protein concentrations were measured using SoftMax Pro 5.44 at a wavelength of 560 nm.

### DNA-damage induction using ionizing radiation and chemical agents

Irradiation with the corresponding dose was performed using the GammaCell 40 from Theatronics. Cisplatin (Teva GmbH) treatment with the indicated dose was done by adding the agent into the culture medium for 18 h then the culture medium was exchanged with fresh and cisplatin-free medium. Olaparib (Selleckchem) was added to the culture medium and left throughout the experiment. Wild-type (WT) or ID3-KO AID-DIvA cells were treated with 300 nM 4-hydroxytamoxifen (4OHT) for 4 h to induce AsiSI localization into the nucleus and generation of DNA double-strand breaks.

### Nuclear lysate extraction and immunoprecipitation (IP)

Adherent cells were washed with cold PBS, trypsinized, and centrifuged at 1000 × g for 5 min at 4°C. Pellet was then resuspended in cell lysis buffer (10 mM HEPES, pH 7.5, 1.5 mM MgCl_2_, 10 mMKCl) supplemented by 1× protease inhibitor and snap-frozen in liquid nitrogen. The frozen suspension was thawed and 1% NP-40 added, then centrifuged at 1500 × g for 15 min at 4°C. The supernatant (cytosolic lysate) was collected. The pellet (nuclei) was washed with cell lysis buffer and centrifuge at 1500 × g for 10 min at 4°C. The pellet (nuclei) was resuspended in nuclei extraction buffer (300 mM NaCl, 20 mM HEPES, pH 7.5, 3 mM MgCl_2_, 20 mMKCl), 1 μl benzonase (DNase) was added and incubate 30 min at RT. Then centrifuged at the highest speed for 15 min at 4°C. The supernatant contained nuclear lysate. The protein concentration was measured using BCA test. For IP 1.5–2 mg of nuclear lysate were filled up with nuclei extraction buffer up to 500 μl. 50 μl of Flag-magnetic beads (prewashed with extraction buffer) were added and incubated overnight at 4°C with rotation. In case of using protein A/G magnet beads, beads were pre-blocked overnight with 0.1% BSA in PBS, while incubating the nuclear lysate with 2–5 μg of the antibody overnight at 4°C with rotation. The pre-blocked beads were added to the lysate–antibody mix and incubated 3 h at 4°C with rotation. Beads were washed three times with TBST followed by a wash with water. Beads were subjected to preparation for mass spectrometry or resuspended in Laemlli buffer (supplemented with 10% β-mercaptoethanol) and incubated 10 min at 95°C then loaded on SDS gel.

### Mass spectrometry sample preparation and data acquisition

After a wash with water, Flag-magnetic beads were then conditioned in 50 mM ammonium bicarbonate NH_4_HCO_3_. Samples were subjected to reduction with DTT 7 mM final at 55°C for 30 min, followed by alkylation with iodoacetamide 12 mM at RT for 40 min in the dark. The reaction was quenched with DTT and proteins were digested on beads with a trypsin/LysC mix (Promega, V5071) at 37°C for 16 h. Digested peptides were desalted with 2 μl of SP3 para-magnetic beads as previously described (23–25). Peptides were eluted in 0.1% trifluoroacetic acid (TFA) in H_2_O, loaded on a trap column (PepMap100 C18 Nano-Trap 100 μm × 2 cm) and separated over a 25 cm analytical column (Waters nanoEase BEH, 75 μm × 250 mm, C18, 1.7 μm, 130 Å,) using the Thermo Easy nLC 1200 nanospray source (ThermoEasynLC 1200, Thermo Fisher Scientific). Solvent A was water with 0.1% formic acid and solvent B was 80% acetonitrile, 0.1% formic acid. During the elution step, the percentage of solvent B increased in a linear fashion from 3% to 8% in 4 min, then increased to 10% in 2 min, to 32% in 68 minutes, to 50% in 12 min and finally to 100% in a further 1 min and went down to 3% for the last 11 min. Peptides were analyzed on a Tri-Hybrid Orbitrap Fusion mass spectrometer (Thermo Fisher Scientific) operated in positive (+2 kV) data-dependent acquisition mode with HCD fragmentation. The MS1 and MS2 scans were acquired in the Orbitrap and ion trap, respectively with a total cycle time of 3 s. MS1 detection occurred at 120 000 resolution, AGC target 1E6, maximal injection time 50 ms and a scan range of 375–1500 *m*/*z*. Peptides with charge states 2–4 were selected for fragmentation with an exclusion duration of 40 s. MS2 occurred with CE 33%, detection in topN mode and scan rate was set to Rapid. AGC target was 1E4 and maximal injection time allowed of 50 ms. Data were recorded in centroid mode.

### Mass spectrometry data processing, analysis and visualization

RAW data were processed with Maxquant software (1.5.1.2) including the Andromeda search engine (26,27). Peptide identification was performed using *Homo sapiens* Uniprot database concatenated to a database containing protein sequences of contaminants (canonical and isoform). Default parameters of Maxquant were used with the following modifications: digestion by Trypsin/P and LysC, default variable modification (methionine oxidation and N-terminal acetylation), cytosine carbamidomethylation as a fixed modification. The Instrument set Orbitrap (with precursor tolerance 20 ppm, MS tolerance 0.5Da). FDR was set to 1% at both protein and peptide levels. Match between runs option was enabled, label-free quantification (LFQ), and iBAQ calculated. For further protein analysis, Perseus free software was used (28). Potential contaminants, reverse proteins, and proteins only identified by sites were removed and only proteins identified with at least one unique peptide in both biological replicates were considered for further analysis. Missing values in the untreated samples were replaced with fixed value corresponding to the lower LFQ log10 value of that experiment. Two-sided t-test statistics were used for the generation of the volcano plots based on LFQ log_10_ values of expressed proteins. FDR was 0.05 and S0 constant was 0.1. Pathway enrichment analysis was done using the Metascape resource (29).

### Clonogenic survival assays

Survival assays were performed as described in ref. (30). Briefly, 500 cells were seeded into 6-well plates 48h following siRNA transfection and incubated overnight at 37°C. Cells were then irradiated or treated by cisplatin or olaparib. Plates were incubated at 37°C, 5% CO_2_ and left for 10–14 days. The medium was removed, and the colonies were fixed using 70% ethanol and stained using 1% crystal violet. The colonies were finally counted. The data are presented as the mean ± SEM value in three independent experiments. One-way ANOVA with Bonferroni's Multiple Comparison Test was performed to compare pairs of siCTR and siID3 at the indicated doses.

### DSB repair reporter assay

5 × 10^5^ U2OS-DR or U2OS-EJ5 cells were seeded in T-25 flasks and transfected with 25 nM of four pooled siRNAs against either ID3, NBS1, RAD50, RECQL, MDC1, XRCC4 or RAD51, or a non-targeting control. After 24 h, the medium was exchanged. Twenty-four hours later, the cells were transfected with 2 μg of plasmid DNA (expression constructs for I-SceI or GFP from Addgene) and incubated in an antibiotic-free medium. After 24 h, the medium was exchanged again, and incubated for another 24 h. The cells were then harvested and resuspended in 1 ml PBS, then were kept on ice until GFP expression is measured in the BD FACSCanto II (BD Biosciences) using the BD FACSDiva Software (BD Biosciences). Results were normalized to transfection efficiency and control siRNA treatment. The data are presented as the mean ± SD value in three independent experiments. One-way ANOVA with Tukey's multiple comparison test was performed.

### Immunofluorescence analysis

2 × 10^5^ cells were seeded in duplicates onto comet slides (R&D systems), incubated overnight then irradiated with 2 Gy. Cells were fixed after the indicated time points in 4% paraformaldehyde for 20 min, permeabilized in 0.15% PBS–Triton X100 for 15 min, and blocked for 30 min (1% BSA and 0.15% glycine in PBS). 50 μl primary antibody is added onto each spot in the indicated dilutions and incubated overnight at 4°C. Slides were washed in PBS and permeabilized for 10 min each before blocking for 10 min. Next, 50 μl secondary antibody were added per spot in the indicated dilutions. Slides were incubated for 45 min at RT in dark and subsequently washed for 10 min in PBS and permeabilized for 5 min. DNA is stained using bis-Benzimide (Sigma Aldrich) in Tris–HCl for 3 min. The mounting medium Vectashield (Vector Laboratories) was added before sealing with a cover slide. Fluorescent images were taken by using the Zeiss Axioplan 2 imaging microscope and foci were automatically counted by Metafer4 system with a magnification of 400×. One thousand cells were counted. The data are presented as the mean ± SEM value in three independent experiments. One-way ANOVA with Bonferroni's multiple comparison test was performed to compare the indicated pairs.

### Extraction of chromatin fractions

Cells were collected by scraping the cells off the flask surface in ice-cold PBS, centrifugation at 1000 × g and 4°C for 5 min. The cell pellet was resuspended in lysis buffer (10 mM HEPES, pH 7.6, 10 mM KCl, 0.05% NP40, freshly supplemented with phosphatase/protease inhibitor cocktail, Roche) and incubated on ice for 30 min. Cells were pelleted by centrifugation at 13 200 rpm and 4°C for 10 min. The supernatant is collected (cytoplasmic fractions). Then the pellet (nuclei) is resuspended in low salt buffer (10 mM Tris–HCl, pH 7.5, 3 mM MgCl_2_, 1% Triton X-100 and supplemented with phosphatase/protease inhibitor cocktail, Roche) and incubated for 15 min on ice. Samples were centrifuged and the supernatant was collected (nuclear soluble fractions). The pellet is resuspended in 0.2 M HCl and incubated for 20 min on ice. Samples were centrifuged and the supernatant is transferred into a new pre-chilled tube (chromatin fractions). The solution is afterward neutralized with 1 M Tris–HCl, pH 8.5.

### Chromatin immunoprecipitation and qPCR (ChIP-qPCR)

U2OS-DR cells were treated with DMSO or 10μM ATM inhibitor (KU55933, Calbiochem) 1h before being transfected with ISceI expressing vector to introduce a DSB, then incubated 8h. Wild-type (WT) or ID3-KO AID-DIvA cells were treated with 300 nM 4-hydroxytamoxifen (4OHT) for 4 h to induce AsiSI localization into the nucleus and generation of DSBs (19). Cells were cross-linked by incubating the adherent cells with formaldehyde (1%) in PBS at 37°C for 10 min then glycine (2.5 M) was added to stop the crosslinking reaction and incubated at 37°C for 10 min. Cells were pelleted (1000 g, 5 min, 4°C), resuspended and washed in 2 ml ice-cold PBS (freshly supplemented with phosphatase/protease inhibitors). Cells were pelleted (3000 g, 5 min at 4°C) and resuspended in 1 ml nuclei preparation (10 mM HEPES at pH 8, 85 mM KCl, 0.5% NP-40) buffer supplemented with protease/phosphatase inhibitors (2×) then transferred into Adaptive Focused Acoustics (AFA) Covaris tube for short sonication using Covaris M220 sonicator (Peak power 40%. Duty factor 2.5, 3–6 min) to isolate the nuclei. Nuclei were pelleted (3000 g, 5 min at 4°C). The nuclei pellet was resuspended in 1 ml shearing buffer (10 mM Tris–HCl at pH 8, 0.1% SDS, 1 mM EDTA) and transferred to a new AFA tube for chromatin shearing (Peak power: 75%. Duty factor 10. Cycle/Burst: 200. For 15 min) to obtain an average fragment size of 200–300 bp. An aliquot of sheared chromatin was taken for de-crosslinking and to check the fragment size. DNA measurement was done using Qubit, then 1 μl of the purified DNA was loaded onto BioAnalyzer chip. Depending on the DNA concentration, 50 μg of the sheared chromatin was prepared and obtained in 500 μl ChIP dilution buffer (20mM HEPES, 0.1% SDS, 1% Triton X100, 150 mM NaCl, 1mM EDTA, 0.5mM EGTA). About 2–5 μg of antibody were added and incubated on a rotator overnight at 4°C. Magnetic beads (ChIP Protein A magnetic beads, Cat. #16-661, Merck Millipore or ChIP-Grade Protein G Magnetic beads, Cat. #9006, cell signaling) were washed and incubated in 0.1% BSA in PBS on the rotator overnight at 4°C. Pre-blocked magnetic beads were added to the antibody–chromatin mixture and incubated for 3 h at 4°C on a rotator. Beads were washed with wash buffer 1 (20 mM HEPES, 0.1% SDS, 1% Triton X-100, 1 mM EDTA, 0.5 mM EGTA, 150 mM NaCl, 0.1% sodium deoxycholate), then wash buffer 2 (20 mM HEPES, 0.1% SDS, 1% Triton X100, 1 mM EDTA, 0.5 mM EGTA, 500 mM NaCl, 0.1% sodium deoxycholate) and with buffer 3 (20 mM HEPES, 0.1% SDS, 1% Triton X100, 1 mM EDTA, 0.5 mM EGTA, 250 mM LiCl, 0.5% sodium deoxycholate, 0.5% NP-40). Finally, beads were washed 2× with ice-cold 10 mM Tris–HCl, pH 8 to remove detergents or salts. Beads were incubated with Elution buffer (10 mM Tris–HCl, pH 8, 5 mM EDTA, 300 mM NaCl, 0.5% SDS) supplemented with proteinase K and incubated for 2 h at 55°C and 8 h at 65°C then treated with RNAse for 20 min at 37°C and stored at 4°C. DNA was then eluted and purified using Ampure beads (Agencourt AMPure XP, Cat. #A63880, Beckman Coulter) at room temperature (with ratio 1:1.2). Then washed twice with 80% ethanol, dried for 10 min and finally eluted in dH_2_O at room temperature. For qPCR, proximal (80bps) or distal (800bps) primers for the indicated DSBs and primers for the promoters of the indicated DNA repair genes were used (Supplementary Table S8, (19)), qPCR was performed using primaQUANT™ CYBR green kit (Steinbrenner Laborsysteme).

### RNA isolation and gene expression analysis by real-time qPCR

RNA was extracted using the TRIzol RNA Isolation Reagents (Thermofisher) according to the manufacturer's instructions. Briefly 5 × 10^5^ cells were washed carefully with PBS and lysed using of Trizol followed by the addition of chloroform. Samples were vortexed thoroughly and left at room temperature for 3 min then centrifuged at 12 000 × g and 4°C for 15 min for phase separation. The upper aqueous phase containing RNA was carefully transferred to a new Eppendorf tube. Isopropanol was added with proper mixing and incubated at room temperature for 10 min, then centrifuged at 12 000 × g and 4°C for 10 min to precipitate RNA. RNA pellet was washed twice with 75% ethanol and resolved in RNase-free water. cDNA is reverse transcribed using the SuperScript™ III First-Strand Synthesis System (Thermofisher) and random priming with hexamers. RT-qPCR is performed using primaQUANT™ CYBR green kit (Steinbrenner Laborsysteme) and intron-spanning primers from Sigma (Supplementary Table S8). Melting Curves are measured using the Lightcycler 480 II from Roche. The gene expression is normalized onto the housekeeping genes and reflects the relative percentage of expression towards the housekeeping genes. The data are presented as the mean ± SD value in independently repeated experiments. One-way ANOVA with Dunnett's multiple comparison test was performed to compare all to the WT untreated.

### RNA purification and gene expression by RNA-seq

RNA is extracted from AID-DIvA cells (*n* = 4 from WT or ID3-KO and *n* = 2 from rescue cells) using the TRIzol RNA Isolation Reagents (Thermofisher). RNA is then purified using the RNeasy Mini spin columns kit (Qiagen) according to manufacturer's instructions. The integrity of the extracted RNA was analyzed by Agilent 2100 Bioanalyzer using Agilent RNA 600 Nano Kit according to the manufacturer's protocol. Sequencing libraries were prepared by the Genomics and Proteomics Core Facility (DKFZ, Heidelberg) from total RNA using the Illumina TrueSeq Stranded Total RNA Library Prep Kit according to the manufacturer's instructions. Multiplexes of four samples were sequenced in a paired-end setting (100 bp) on an Illumina NovaSeq 6000 machine for sequencing. Data were processed by the Omics IT and Data Management Core Facility (DKFZ, Heidelberg), using the Roddy RNA-seq Workflow. Default parameters were used unless mentioned otherwise. Sequences were aligned to the human reference genome (hg19/GRCh37) by applying the software STAR (31). Gene counts were generated with featureCounts (32) and the gene annotation v.29 lift 37. For the identification of differentially expressed genes, the R library DESeq2 was used (33). The date of sample preparation was included as a batch in the design formula. Genes with an absolute log_2_ fold-change >0.5 and an adjusted *P*-value <0.05 were defined as significantly differentially expressed genes (DEG). Gene set enrichment analysis (GSEA) against custom gene sets was performed with the R package clusterProfiler (34). For this, genes were ordered according to their log_2_ fold change and the fgsea algorithm was applied (Sergushichev A (2016), http://biorxiv.org/content/early/2016/06/20/060012). Custom gene sets representing DNA damage repair pathways were required from (35).

### Assay for transposase-accessible chromatin using sequencing (ATAC-seq)

ATAC-seq libraries from WT and ID3-KO (AID-DIvA cells) samples (*n* = 3), with and without irradiation, were prepared according to the Omni-ATAC protocol (36). 50 000 viable cells were pelleted at 500 × g for 10 min and washed in PBS. Subsequently, nuclei were isolated using ice-cold lysis buffer (0.01% digitonin, 1% NP40 [Genaxxon Bioscience], 0.1% Tween-20). Nuclei were resuspended in ATAC resuspension buffer (10 mM Tris–HCl pH 7.4, 10 mM NaCl, 3 mM MgCl_2_) and pelleted at 500 g for 10 min. Nuclei were then resuspended in 2× transposition buffer (20 mM Tris–HCl pH 7.6, 3 mM MgCl_2_, 20% dimethyl formamide) and the tagmentation reaction was performed by adding 2.5 μl of Tagment DNA Enzyme 1 (Illumina). The mixture was rotated at 1000 rpm for 30 min at 37°C. DNA was further purified using 140 μl of AMPure XP beads (Beckman Coulter), 20 μl of 5 M guanidinium thiocyanate (Sigma-Aldrich). Libraries were amplified in two PCR reactions by adding 25 μl of NEBNext High Fidelity 2× Master Mix (NEB), 0.8 μl of 10 μM Custom Nextera PCR Primer 1, and 0.8 μl of 10 μM Custom Nextera PCR Barcode to 25 μl of the transposed DNA. The first amplification used the following PCR program: 5 min at 72°C, 30 s at 98°C, 5 cycles of 10 s at 98°C, 30 s at 63°C, and 1 min at 72°C, and, finally, 1 min at 72°C. The number of necessary additional cycles to reach sufficient DNA amplification was determined using 5 μl of the pre-amplified PCR mixture, Sybr Green I nucleic acid gel stain (Thermo Fisher Scientific) and a light cycler instrument (Roche) by applying the following program: 30 s at 98°C, 20 cycles of 10 s at 98°C, 30 s at 63°C, 1 min at 72°C, and, finally, 1 min at 72°C. Based on the quantitative PCR results, additional PCR cycles were applied to the remaining pre-amplified mixture. The final libraries were purified with a two-sided size selection applying 0.5× and 1.4× of AMPure XP beads (Beckman Coulter). Beads were washed shortly with 80% ethanol and kept on a magnetic rack for 10 min to dry. Finally beads were resuspended in 1× EB buffer (Qiagen) and put back on a magnetic rack until bead suspension has cleared, then the supernatant was transferred (DNA eluate) to new 8-well strip. The concentration of the library was determined using the Qubit dsDNA HS Assay Kit (Thermo Fisher Scientific). Quality control was performed on a Bioanalyzer station using the Agilent High Sensitivity DNA Kit. Sequencing was performed at the DKFZ Genomics and Proteomics Core Facility using the High Seq 2000 v4 Paired-End 125 bp.

Sequencing reads were adapter and quality trimmed by deploying Trim Galore v. 0.4.4: (http://www.bioinformatics babrahamacuk/projects/trim_galore) in conjunction with Cutadapt v. 1.14 (37) and the non-default parameters ‘–paired’, ‘–nextera’, ‘–length_1 35’, and ‘–length_2 35’. Trimmed reads were aligned against the Genome Reference Consortium Human Build version 37 by means of Bowtie2 v. 2.2.6 (38) using the ‘–very-sensitive’ flag and a maximal insertion length of 2500 bp. Alignments belonging to the same lane-multiplexed library were pooled using SAMtools mergev. 1.5 (39). PCR duplicates were removed using Picard MarkDuplicates v. 2.17.4 (http://broadinstitutegithubio/picard/). Reads that did not align in a proper pair as well as mappings with a quality below 20 on the Phred scale were removed by means of SAMtools view. As previously demonstrated by Adey *et al.* (40), the binding site of a Tn5 transposase homodimer consists of a 9 bp central region, in which the transposition event occurs, and two 10 bp flanking regions. Thus, fragments resulting from tagmentation cannot be smaller than 38bp. all alignment below this size were discarded. Read ends were adjusted to represent the midpoint of the transposition event as previously described by (41). To smooth the accessibility signal, a 73 bp window was centered on each transposition midpoint and the resulting tag coordinates were used in all downstream analyses. Peak calling was carried out using MACS2 callpeak v. 2.1.0.20140616 (42) with a *q*-value cutoff of 0.05 and the non-default parameters ‘–nomodel’, ‘–broad’, ‘–gsize 2809561002’, and ‘–keep-dup all’. The analysis procedure has been implemented as a fully containerized workflow using the Common Workflow Language v. 1.0 (https://doiorg/106084/m9figshare3115156v2) and is publicly accessible (43).

Differential accessibility analysis was performed using the DiffBind R package (R package version 2.12.0) (44). A common peak set was identified by the presence of a peak in at least two samples. Differential analysis was performed using the edgeR method (45). Regions with an FDR <0.05 and an absolute log_2_ fold change >1 were considered as differentially accessible. Further annotation of all differentially accessible regions was performed with R package ChIPseeker (R package version 1.20.0) (46).

To assess differential transcription factor activity, diffTF was applied to the ATAC-seq data set (47). diffTF (version 1.3.3) was used in analytical mode comparing ID3-KO versus WT samples, with and without irradiation. Human transcription factors with in silico predicted transcription factor binding sites based on the HOCOMOCO 10 database were used as a reference (48). Mean target gene expression was evaluated for transcription factors with an adjusted *P*-value <0.05, by averaging the expression log_2_ fold change for each gene, with the respective transcription factor motif <3000 bp away from the transcriptional start site. Annotation of the transcription factor binding sites was performed as described above for DARs. Profile plots were generated with the R library peakseason (https://github.com/PoisonAlien/peakseason). *P*-values were determined with a t-test after the normal distribution was shown with a Shapiro-Wilk test (*P* < 0.05).

### Correlation of ID3 expression and DNA repair gene expression using TCGA data of cancer patients

The harmonized HT-Seq counts were downloaded using TCGAbiolinks (49) from Genomic Data Commons (GDC) (https://gdc.cancer.gov/), and only patients with ‘primary tumor’ status were used. Genes with <10 counts were filtered, and log_2_ counts per million (log_2_ (CPM + 1)) were calculated, followed by trimmed mean of *M* values (TMM) normalization (50). Gene ontologies of DOUBLE STRAND BREAK REPAIR VIA BREAK INDUCED REPLICATION (GO:0000727), RECOMBINATIONAL REPAIR (GO:0000725), REGULATION OF DOUBLE STRAND BREAK REPAIR VIA HOMOLOGOUS RECOMBINATION (GO:0010569), and REPLICATION BORN_DOUBLE STRAND BREAK REPAIR VIA SISTER CHROMATID EXCHANGE (GO:1990414), were downloaded from the reference MSigDbdatabase (v6) (51), (https://www.gsea-msigdb.org). Single sample gene set enrichment scores were estimated using the GSVA package (52), for the four gene ontologies and were further used to perform hierarchical clustering followed by dynamic tree cut (53) to estimate the number of groups per tumor type. For bioinformatics analysis, unless stated otherwise, all group comparisons were performed using Kruskal–Wallis and Wilcoxon rank-sum tests, and all reported *P*-values were adjusted using the Benjamini–Hochberg procedure. Pearson correlation coefficients were estimated for all gene–gene correlations. All analyses were run in R, version 3.6.1, (https://cran.r-project.org/) and Bioconductor version 3.9 (https://bioconductor.org/). All graphical representations were generated using *pheatmap ggplot2*, *corrplot*, *ggpubr*, and *RcolorBrewer*.

## RESULTS

### ID3 interacts with MRN complex subunits and the RECQL helicase in response to DNA damage.

**Figure 1.**
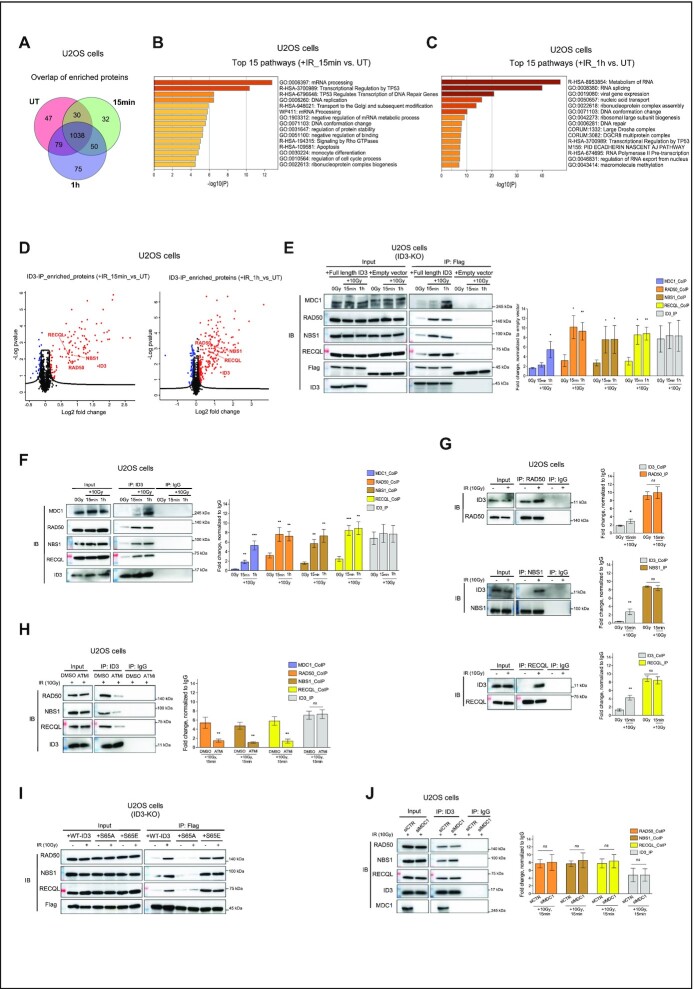
ID3 interacts with DNA repair proteins after IR. ID3-KO cells expressing Flag-tagged ID3 were irradiated with 10 Gy and harvested at 15 or 60 min after irradiation. (**A**) Venn diagram showing the mass spectrometry-identified interaction peptides among unirradiated (UT) and irradiated samples after 15 and 60 min. (**B**) Pathway enrichment analysis using the interaction proteins 15 min after irradiation. Analysis was done using Metascape resources (Ref.# 71). (**C**) Pathway enrichment analysis using the interaction proteins 1h after irradiation. Analysis was done using Metascape resources (Ref.# 71). (**D**) Volcano plot showing identified ID3 interaction candidates, identified proteins are highlighted in red. Y-axis represents *t*-test statistics of log-10 LFQ intensities. (**E**) IP of Flag-ID3 using Flag-beads followed by Western blot to validate co-IP of the identified DNA repair proteins interacting with ID3 (Representative Western blot, left panel) and a bar plot for the quantification of three independent Western blots (right panel, mean ± SD). (**F**, **G**) Western blot to confirm interaction of endogenous ID3 to RAD50, NBS1 and RECQL (left panels) and bar plot showing the quantification of three independent Western blots (right panels, mean ± SD). (**H**) Western blot of the co-IP of RAD50, NBS1 and RECQL to ID3 after treatment either with DMSO or 10 μM ATMi prior to irradiation (representative Western blot, left panel) and a bar plot of the quantification of three independent Western blots (right panel, mean ± SD). **(I)** Western blot of the co-IP of RAD50, NBS1 and RECQL to WT-ID3, phospho-dead ID3 (S65A) and phospho-memic ID3 (S65E). (**J**) Western blot of the co-IP of RAD50, NBS1 and RECQL to endogenous ID3 upon knockdown of MDC1 prior to irradiation (representative Western blot, left panel) and a bar plot for the quantification of three independent Western blots (right panel, mean ± SD). Student's *t* test was used. Statistical significance is presented as: * P < 0.05, ** P< 0.01, ***P< 0.001, ****P < 0.0001, ns = not significant.

To explore possible protein interactions with ID3 after DNA damage, we first generated CRISPR-Cas9-mediated ID3 knockout cells (U2OS cells) and transduced them with GFP-Flag-tagged-ID3 expressing vector or an empty vector as a control (Supplementary Figure S1A). These cells with ID3 expression rescue were either left untreated or irradiated with 10 Gy or subsequently harvested after 15 min or 1 h. Although separation of nuclear lysates displayed that flag-ID3 was more abundant in the cytosolic fraction which is in contrast to the fractionation of the endogenous ID3 (Supplementary Figure S1B and D), the immunoprecipitation of flag-ID3 from nuclear fractions successfully showed high enrichment of flag-ID3 (Supplementary Figure S1C). Enriched ID3 was subjected to label-free quantitative mass spectrometry. Using cutoffs for fold change >0.5 and adjusted *P*-value <0.05, data analysis identified1194 protein interactions occurring in untreated cells, while 1150 and 1242 interactions were in cells collected 15 min or 1 h after IR, respectively (Figure 1A and Supplementary Tables S1-4). We then performed Gene Ontology (GO) enrichment analyses using the Metascape resource (29). Among all the three conditions a strong enrichment was observed for pathways involved in metabolism of RNA, translation, cell cycle and transcription regulation (Supplementary Figures S1F and G, and S2A). Interestingly, DNA repair is present among the top 25 significant enriched pathways only in the irradiated cells (Supplementary Figure S1G). Since we were interested in DNA damage-induced interaction proteins, we focused on those protein interactions that were highly enriched in IR treated cells over the untreated (Supplementary Tables S5 and S6), and performed Gene Ontology (GO) enrichment analyses. A strong enrichment was observed for RNA metabolism, transcription regulation TP53 pathways, cell cycle regulation as well as for DNA replication and repair (Figure 1B and C). By exploring the components of both the DNA repair and transcription regulation TP53 pathways, we identified GO:0006302 double-strand break repair which includes several interaction candidates of ID3 which have known roles in DSB repair (Supplementary Table S7). These include two members of the MRN complex, NBS1 and RAD50 as well as RECQL, a member of the ATP-dependent RecQ DNA helicase family, (Figure 1D, and Supplementary Figure S2B and C).

Western blot analyses confirmed co-immunoprecipitation of Flag-ID3 with NBS1, RAD50 and RECQL post-IR and earlier than MDC1 (Figure 1E). These interactions were confirmed by performing immunoprecipitation of the endogenously expressed ID3 (Figure 1F and G) and they were ATM-dependent, as inhibition of the ATM kinase by the specific inhibitor KU55933 reduced ID3 co-immunoprecipitation with all three proteins (Figure 1H and Supplementary Figure S1E). Since ATM is known to phosphorylate ID3 at Serine 65 (S65) (18), we compared the interaction of the identified proteins witheither wild-type ID3, phospho-dead (S65A) or phospho-mimic form (S65E). Our Western blot results demonstrate interaction between the three repair proteins and phospho-mimic form (S65E) before and after IR, while this interaction was lost in the case of phospho-dead form (S65A) (Figure 1I). This indicates that ID3 associates with the DNA repair machinery after irradiation and is interacting with both the MRN complex and RECQL in an ATM-dependent manner. As this ATM-dependent phosphorylation of ID3 at S65 was described as crucial for interaction between ID3 and MDC1, we tested whether the interaction of ID3 with RAD50, NBS1 or RECQL requires MDC1. Depletion of MDC1 did not influence the interaction of the newly identified repair factors with ID3 (Figure 1J), suggesting that ID3 is able to interact with RAD50, NBS1 and RECQL independent of its reported interaction with MDC1. Our mass spectrometry data and the additional co-immunoprecipitation of other DNA repair proteins such as CtIP or RAD51 showed no direct interaction to ID3, thus confirming that the identified interactions are specific and not random due to abundance of ID3 in the nucleus (Supplementary Figure S2D).

### ID3 is required for DSB repair and cooperates with the MRN complex and RECQL

To further elucidate the mechanism of ID3 in DNA repair, ID3 wild-type (WT) cells and CRISPR-Cas9-mediated ID3 knockout (ID3-KO) cells were treated with IR (2 Gy) and analyzed for their colony formation ability and number of γH2AX *foci*. ID3-KO cells showed higher cellular sensitivity to IR and more residual γH2AX *foci* compared to WT cells (Supplementary Figure S2E and F). Reintroduction of GFP-Flag-tagged ID3 to the knockout cells rescued their DSB repair efficiency following IR exposure (Supplementary Figure S2G).

**Figure 2.**
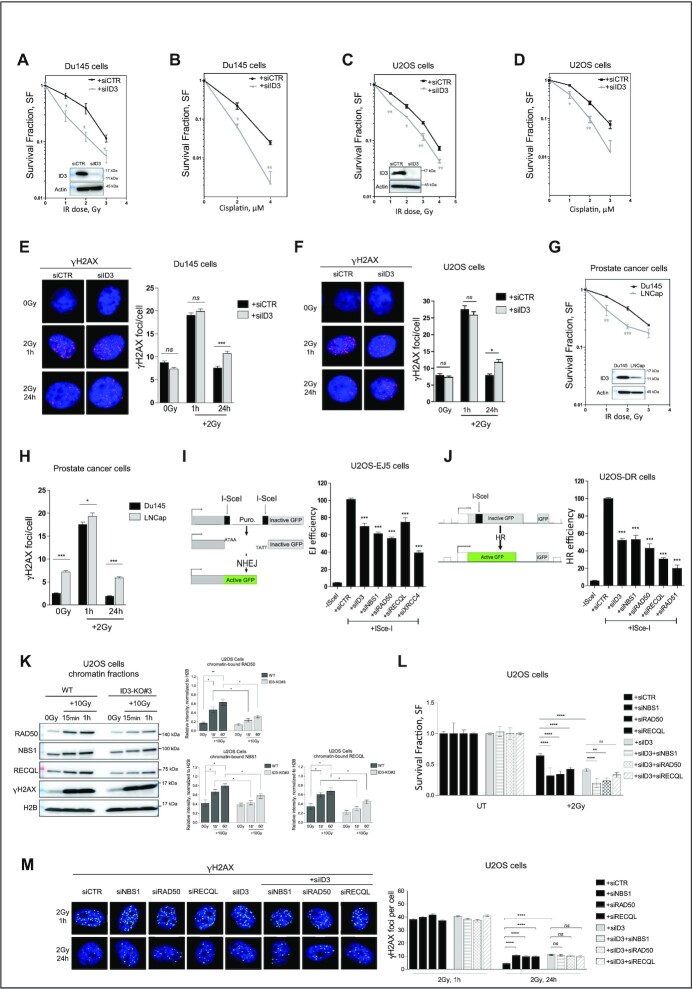
ID3 is required for cell survival and DSB repair. (**A–D**) Clonogenic survival assay of Du145 and U2OS cells transfected either with control siRNA (siCTR) or a pool of four siRNAs targeting ID3 (siID3) and treated with the indicated dose of ionizing radiation or concentrations of cisplatin. *n* = 3 independent experiments;data are presented as mean ± SEM, one-way ANOVA with Bonferroni's multiple comparison test. (**E**, **F**) Representative micrographs and quantification of IR-induced γH2AX foci in Du145 and U2OS cells, ca. 500 cells were counted at indicated time points. *n* = 3 independent experiments; data are presented as mean ± SEM, one-way ANOVA with Bonferroni's multiple comparison test. (**G**) Clonogenic survival assay of prostate cancer cell lines with different ID3 expression levels treated with the indicated doses of ionizing radiation. *n* = 3 independent experiments; data are presented as mean ± SEM, One-Way ANOVA with Bonferroni's multiple comparison test. (**H**) Quantification of IR-induced γH2AX foci in prostate cancer cell lines with different ID3 expression levels treated with 2 Gy, ca. 500 cells were counted at the indicated time points. *n* = 3 independent experiments; data are presented as mean ± SEM, One-Way ANOVA with Bonferroni's multiple comparison test. (**I**, **J**) NHEJ efficiency measured in U2OS-EJ5 cells and HR efficiency measured in U2OS-DR cells using the indicated siRNAs, respectively. *n* = 3 independent experiments; data are presented as mean ± SD, one-way ANOVA withTukey's multiple comparison test. (**K**) Western blot of the chromatin-bound fractions of RAD50, NBS1 and RECQL in U2OS cells (Representative Western blot, left panel) and a bar plot of the quantification of three independent Western blots (right panel, mean ± SD). (**L**) Clonogenic survival assay of U2OS cells transfected with the indicated siRNAs and treated with 2Gy of ionizing radiation. *n* = 3 independent experiments; data are presented as mean ± SEM, one-way ANOVA with Bonferroni's multiple comparison test. (**M**) Representative Micrographs and quantification of IR-induced γH2AX foci in U2OS cells transfected the indicated siRNAs, ca. 500 cells are counted at indicated time points. *n* = 3 independent experiments; data are presented as mean ± SEM, one-way ANOVA with Bonferroni's multiple comparison test. Statistical significance is presented as: * P < 0.05, ** *P* < 0.01, *** *P* < 0.001, **** *P* < 0.0001, ns = not significant.

To recapitulate our findings in further cellular models, we depleted ID3 in human prostate cancer cells (Du145) and human osteosarcoma cells (U2OS) using a pool of four different specific siRNAs (Supplementary Table S8). The knockdown efficiencies were confirmed by Western blot (Figure 2A and C). Subsequently, the cells were treated either with IR or cisplatin and analyzed for their colony formation ability. ID3-depleted cells displayed a higher cellular sensitivity to both DNA damaging agents (Figure 2A–D). Furthermore, ID3-depleted cells showed an increased number of residual γH2AX *foci* 24 h after treatment (Figure 2E and F). Together, these results suggest an association of high cellular sensitivity and impaired DSB repair. To consolidate these observations, we used publicly available RNA-seq data of the Broad Institute (https://portals.broadinstitute.org/ccle/page?gene=ID3) to select additional cancer cell lines with low levels of ID3 expression (LnCap and PSN-1) and investigated those by Western Blot. Low ID3 expression in these cells was associated with an increased sensitivity to IR and with an impaired DSB repair (Figure 2G and H, Supplementary Figure S2H and I). ID3 did not form IR-induced *foci* following irradiation (Supplementary Figure S2J). However, ChIP-qPCR analyses showed accumulation of ID3 together with γH2AX close to ISceI-induced DSBs, and this enrichment was dependent on ATM kinase activity (Supplementary Figure S2K).

We further investigated the contribution of ID3 to both NHEJ and HR. Similar to the depletion of the identified repair proteins, knockdown of ID3 decreased the efficiency of both DSB pathways, however HR was more strongly reduced than NHEJ (Figure 2I and J). The knockdown efficiency was confirmed by western blot (Supplementary Figure S2L). We then tested the effect of ID3 loss on the accumulation of NBS1, RAD50 and RECQL within chromatin fractions of irradiated and untreated cells using wild-type U2OS and ID3-KO cells. ID3 silencing reduced the enrichment of NBS1, RAD50 and RECQL to chromatin (Figure 2K). Next, we performed single and double depletion of ID3 together with either NBS1, RAD50 or RECQL (Supplementary Figure S2M) and subsequently monitored cellular survival and formation of γH2AX *foci* following IR. Single depletion of either ID3, NBS1, RAD50 or RECQL displayed a comparable reduction of cell survival and high residual γH2AX *foci*. Simultaneous loss of ID3 and either NBS1 or RAD50 showed a stronger reduction of cell survival after IR compared to the single loss (Figure 2L), while there was no significant difference in the number of residual γH2AX *foci* (Figure 2M). In contrast, no difference in cellular survival or DSB repair was observed when we investigated the simultaneous versus single loss of ID3 and RECQL (Figure 2L and M). These results suggest that ID3 and the MRN complex work via different mechanisms in cell survival after DNA damage, while, with regard to DSB repair, they cooperate with RECQL. On the other hand, the simultaneous depletion of ID3 and MDC1 led to a higher number of residual γH2AX *foci* compared to the single depletions, implying an additive effect (Supplementary Figure S2N).

### ID3 has a major regulatory role in HR.

**Figure 3.**
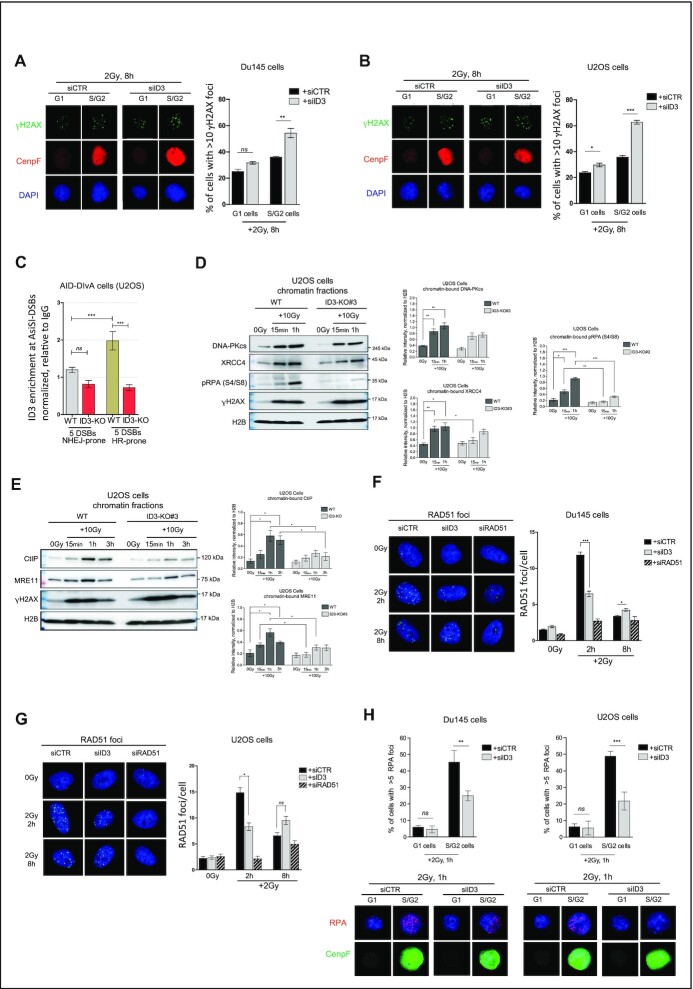
ID3 loss impacts HR. (**A**, **B**) Representative micrographs and quantification of IR-induced γH2AX foci in Du145 and U2OS cells counted in G1 and S/G2 cells using the cell cycle marker CenpF. *n* = 3 independent experiments; data are presented as mean ± SD, one-way ANOVA withTukey's multiple comparison test. (**C**) Enrichment of ID3 at NHEJ-prone and HR-prone DSBs in AID-DIvA cells, measured by ChIP-qPCR. *n* = 3 independent experiments; data are presented as mean ± SEM,Student's *t* test. (**D**) Western blot of the chromatin-bound fractions of DNA-PKcs, XRCC4 and pRPA (S4/S8) (representative Western blot, left panel) and a bar plot of the quantification of three independent Western blots (right panel, mean ± SD), Student's *t* test was performed. (**E**) Western blot of the chromatin-bound fractions of CtIP and MRE11 (representative Western blot, left panel) and a bar plot of the quantification of three independent Western blots (right panel, mean ± SD), Student's *t* test was performed. (**F**, **G**) Representative micrographs and quantification of IR-induced RAD51 foci in Du145 and U2OS cells, respectively, ca. 500 cells were counted at indicated time points. *n* = 3 independent experiments; data are presented as mean ± SEM, Student's *t* test was performed. (**H**) Representative micrographs and quantification of IR-induced RAD51 foci in Du145 and U2OS cells, respectively, ca. 500 cells were counted at indicated time points. *n* = 3 independent experiments; data are presented as mean ± SEM, Student's *t* test was performed. Statistical significance is presented as: * P< 0.05, ** P < 0.01, *** *P* < 0.001, **** *P* < 0.0001, ns = not significant.

Our results obtained from NHEJ and HR reporter assays (Figure 2I and J) suggested a stronger involvement of ID3 in HR as compared to NHEJ. To investigate this further, we monitored the ability to resolve γH2AX *foci* following IR in a cell cycle-dependent-manner, as HR is only performed in S/G2 while NHEJ is used during all cell cycle phases. Eight hours after IR, ID3 depletion resulted in a higher fraction of cells with more than 10 γH2AX *foci*/nucleus in S/G2 versus G1 cell populations (Figure 3A and B). We then used the well-established AID-DIvA cells (19) to study the genome-wide induction of site-specific DSBs. We performed ChIP-qPCR to compare the accumulation of ID3 at five specifically NHEJ-prone and five specifically HR-prone DSBs. We observed higher enrichment of ID3 at HR-prone DSBs compared to NHEJ-prone DSBs, supporting a major role of ID3 in HR (Figure 3C and Supplementary Figure S3A). Furthermore, we examined pathway-specific proteins which act within early steps of NHEJ (DNA-PKcs, XRCC4) and HR (pRPAS4/S8, CtIP and MRE11) for their recruitment to chromatin after IR in WT and ID3-KO U2OS cells. Western blot analyses of ID3-KO cells showed reduced enrichment of XRCC4 15 minutes after IR, while no significant effects on the enrichment of both DNA-PKcs and XRCC4 occurred 1 h after IR (Figure 3D). These results suggest only limited effects of ID3 loss on NHEJ. In contrast, the recruitment of the end resection factors pRPA, CtIP and MRE11 was strongly reduced (Figure 3D and E). DNA damage induction was assessed by γH2AX enrichment in the chromatin fractions at the analyzed time points. The expression of total RPA (Supplementary Figure S3B) and that of several DNA repair proteins in the untreated situation (Supplementary Figure S3C) was not affected by ID3 depletion. Furthermore, analysis of IR-induced RAD51 *foci* formation revealed that ID3 inactivation resulted in a severe reduction of RAD51 loading (Figure 3F and G). In line with these results, PSN1 and LNCap cell lines with low ID3 expression showed reduced RAD51 *foci* formation compared to MIA PaCa-2 and Du145 cells which have high ID3 expression (Supplementary Figure S3E). Cell cycle analyses showed a slight delay in S-phase entry in ID3-depleted cells (Supplementary Figure S3F), which cannot, however, explain the observed effects of ID3 loss on RAD51 foci formation. In addition, we counted RPA *foci* in G1 and S/G2 cells in ID3-depleted cells which showed impaired RPA foci formation in ID3 depleted S/G2 cells (Figure 3H and Supplementary Figure S3D). All in all, these data suggest that HR is the major repair pathway affected by ID3 loss.

So far, our results revealed interactions of ID3 with the MRN complex and RECQL. We next examined whether these interactions regulate HR and RAD51 loading. We therefore monitored HR efficiency and RAD51 foci formation after single and simultaneous depletion of ID3 and NBS1, RAD50, MDC1 or RECQL. Simultaneous knockdown of ID3 and either of the aforementioned factors did not show any further effects on HR efficiency measured by a reporter assay compared to the single ID3 knockdown (Supplementary Figure S3G). Remarkably, simultaneous depletion of ID3 and either NBS1, RAD50 or MDC1displayed lower RAD51 *foci* formation compared tosingle loss of either of them, while no such effect was observed forsimultaneous versus single loss of ID3 and RECQL (Supplementary Figure S3H and I). These data propose that ID3 is regulating RAD51 loading in a different way than the MRN complex,whereas RECQL may mediate the ID3 function to promote RAD51 loading and downstream steps of HR. Next, we investigated in wild-type versus ID3-knockout AID-DIvA cells whether the interaction of ID3 with RECQL affects its accumulation at DSBs that are preferentially repaired *via* HR. ChIP-qPCR analyses revealed that the knockout of ID3 reduced RECQL enrichment at three out of four investigated HR-prone DSBsites (Supplementary Figure S3J).

### ID3 loss leads to a downregulation of HR genes in response to IR

**Figure 4.**
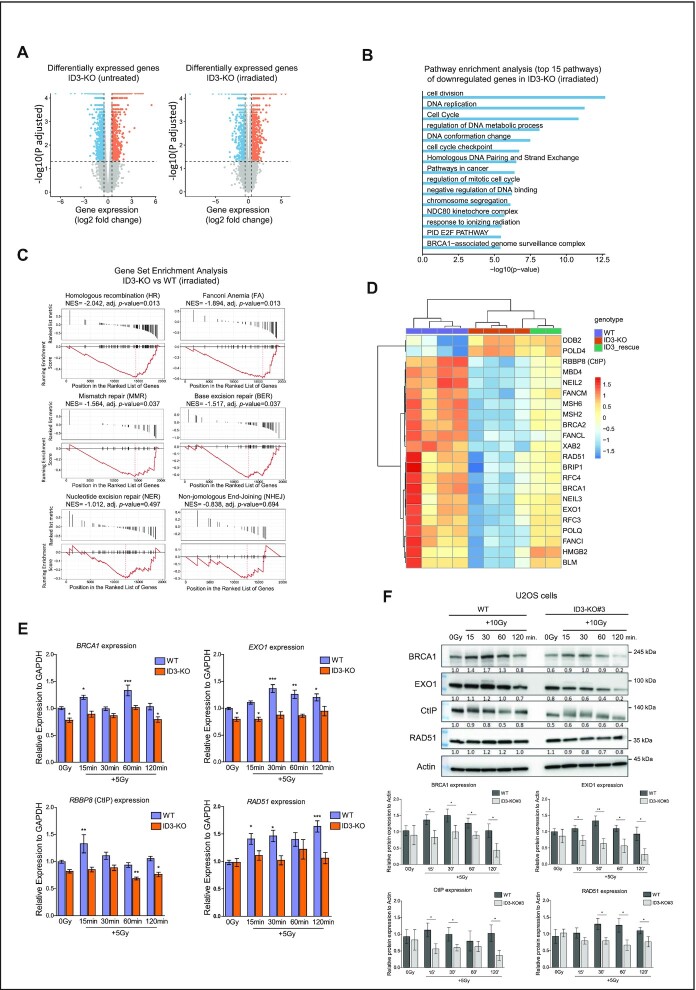
ID3 regulates expression of genes involved in DNA repair in response to IR. (**A**) Volcano plots showing differentially expressed genes in ID3-KO cells; untreated (0 Gy, left) and irradiated cells (5 Gy, right). Measured by RNA-seq (*n* = 4). (**B**) Gene ontology enrichment analysis of the significantly downregulated genes in irradiated ID3-KO cells. (**C**) Barcode plot showing gene set enrichment analysis of DNA repair pathways of differentially expressed repair genes in irradiated ID3-KO cells. NES = normalized enrichment score. (**D**) Heat map showing differentially expressed DNA repair genes with a log_2_ fold change < -0.5 for downregulated genes and > 0.5 for upregulated genes in irradiated WT, ID3-KO and ID3-rescue cells. *n* = 4, 4 and 2 for WT, KO and rescue cells, respectively. (**E**) RT-qPCR expression analysis of *BRCA1, EXO1, RBBP8 (CtIP)* and *RAD51* genes in WT and ID3-KO with IR treatment (5 Gy) at indicated time points, *n* = 5, data presented as mean ± SEM, one-way ANOVA with Dunnett's multiple comparison test to compare all to the WT untreated. (**F**) Western blot showing expression of BRCA1, EXO1, RBBP8 (CtIP), and RAD51 proteins in WT and ID3-KO with IR treatment at indicated time points (representative Western blot, upper panel) and a bar plot of the quantification of three independent Western blots (lower panel, mean ± SD), Student's *t* test was performed. Statistical significance is presented as: * P< 0.05, ** P < 0.01, *** P < 0.001, **** P < 0.0001, ns = not significant.

The proteomic analysis revealed that ID3 interacts not only with DNA repair proteins but also with proteins involved in transcription, which is consistent with the known roles of ID3 in transcriptional regulation. We therefore investigated the impact of ID3 loss on transcription with special focus on genes involved in DNA repair. Our western blot results showed that ID3 loss did not affect the expression of several DNA repair proteins under unperturbed situations (Supplementary Figure S3C). We investigated the effect of irradiation by treating WT, ID3-KO and ID3-KO re-expressing Flag-ID3 cells (rescued cells) with a moderate radiation dose of 5Gy. For this, the cells were collected 15 min after IR, and the RNA was subsequently isolated and sequenced. In irradiated cells, the comparison of gene expression in ID3-KO versus WT cells revealed that 1276 genes were upregulated (adj. *P* < 0.05, log_2_ fold change > 0.5) and 1109 genes were downregulated (adj. *P* < 0.05, log_2_ fold change < -0.5) in ID3-KO cells (Figure 4A, right panel and Supplementary Table S10). GO and pathway analysis of the downregulated genes resulted in enrichment of terms involved in cell cycle regulation, DNA replication, response to radiation, homologous DNA pairing, and the BRCA1-associated genome surveillance, while no DNA repair-related pathways were identified upon analysis of the upregulated genes (Figure 4B and Supplementary Figure S4C). Further enrichment analyses for DNA repair pathway gene sets revealed a negative normalized enrichment score (NES) for all pathways (Figure 4C). However, the changes in HR, Fanconi Anemia (FA), mismatch repair (MMR) and base excision repair (BER) were significant. In contrast the changes in nucleotide excision repair (NER) and NHEJ were not significant. The top two downregulated repair pathwaysin ID3-depleted cells after IR were HR and FA. Further investigation of genes involved in DNA repair pathways revealed downregulation of genes involved in HR and FA, including *EXO1*, *RBBP8(CtIP)*, *FANCM*, *FANCL, BRCA1, BRCA2*, *RAD51*, *POLQ*, *RFC3* and *RFC4* (Figure 4D). Re-introduction of ID3 in the KO cells displayed intermediate expression values of those repair genes compared to WT and ID3-KO (Figure 4D). This shows that the reintroduction of ID3 in KO cells partially rescues the expression changes of these DNA repair genes. The loss of ID3 in untreated cells leads to 1671 differentially expressed genes (DEGs) (Figure 4A, left panel and Supplementary Table S9). Although gene set enrichment analyses (GSEA) did not show any significant enrichment for gene sets representing DNA repair pathways (Supplementary Figure S4A), looking at individual DNA repair genes we identified 7 DNA repair genes which were differentially expressed (adj. *P* < 0.05, absolute log_2_ fold change > 0.5) (Supplementary Figure S4B). Noteworthy, most of the differentially expressed repair genes in the irradiated ID3-KO cells were not altered in the untreated condition. When comparing cells with a loss of MDC1 to those with ID3-depletion, a divergent expression pattern was observed (Supplementary Figure S4D and E). This confirms that loss of ID3 causes alterations independent from MDC1. We further validated the expression of selected repair genes with RT-qPCR and observed that RNA and protein expression of almost all of the tested genes were induced in response to IR in the WT cells, while this induction was missing in ID3-KO cells (Figure 4E and F, and Supplementary Figure S4F and G). Taken together, these results indicate that the absence of ID3 leads to the loss of a transcription regulatory axis responsible for DNA repair gene induction in response to IR.

### ID3 regulates chromatin accessibility of promoters of DNA repair genes involved in HR

**Figure 5.**
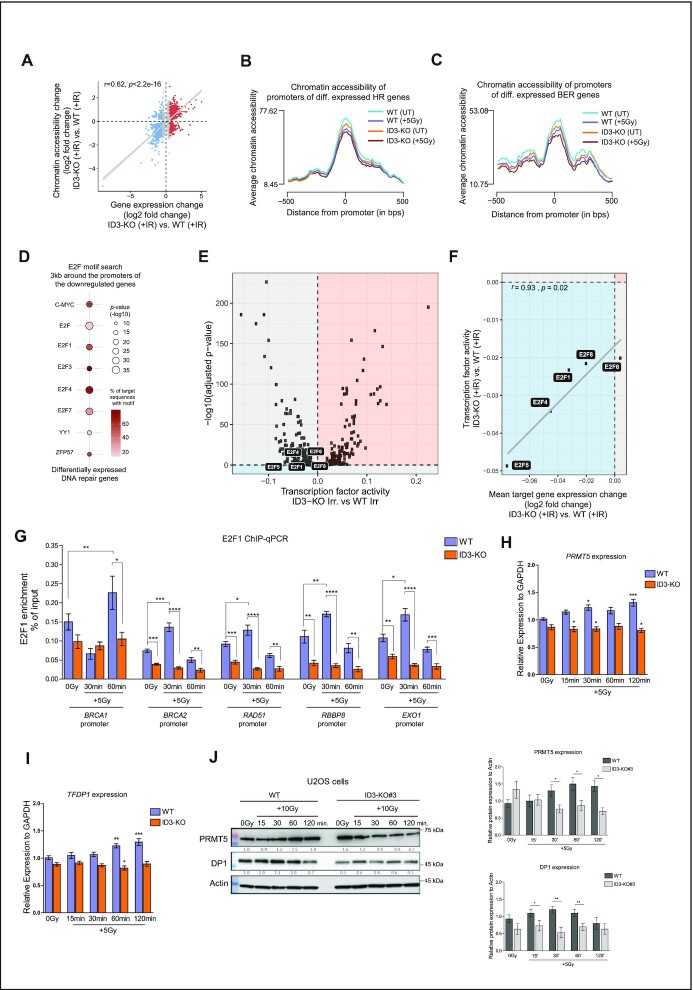
ID3 regulates the chromatin accessibility and activity of E2F1 TF which mediate expression of HR genes in response to IR. (**A**) Scatter plot showing the correlation between differential chromatin accessibility at promoter regions and gene expression in irradiated cells. (**B**, **C**) Mean of the chromatin accessibility at promoter regions of genes involved in HR and BER. *n* = 3 for WT and KO, Student's *t* test. (**D**) Transcription factor binding motif analysis in all downregulated genes in ID3-KO after IR. (**E**) DiffTF analysis showing the differential activity of TFs in irradiated cells, E2F TFs are highlighted. Blue area represents TFs with reduced activity, orange represents TFs with increased activity. (**F**) Correlation between the activity of E2F TFs and the expression of their target genes. (**G**) Enrichment of E2F1 at the promoters of the indicated genes in WT and ID3-KO cells, measured by ChIP-qPCR. *n* = 2 independent experiments; data are presented as mean of six technical replicates from two independents experiments ± SEM, Student's *t* test. (**H**, **I**) RT-qPCR expression analysis of *PRMT5* and *TFDP1* genes in WT and ID3-KO with IR treatment at indicated time points, *n* = 5, data presented as mean ± SEM, one-way ANOVA with Dunnett's multiple comparison test to compare all to the WT untreated. (**J**) Western blot showing expression of PRMT5 and DP1 proteins in WT and ID3-KO with IR treatment at indicated time points (Representative Western blot, left panel) and a bar plot of the quantification of three independent Western blots (right panel, mean ± SD), Student's *t* test was performed. Statistical significance is presented as: * P < 0.05, **P < 0.01, *** *P* < 0.001, **** *P* < 0.0001, ns = not significant.

We analyzed chromatin accessibility in untreated and irradiated ID3-KO and WT samples to explore how ID3 loss can affect the induction of various HR and FA genes in response to IR. Differentially accessible regions (DARs) were annotated to the overlapping or closest genes. A positive correlation between changes in gene expression and chromatin accessibility was observed in ID3-KO versus WT cells both before and after IR (Figure 5A and Supplementary Figure S4H). We observed a reduced accessibility in irradiated ID3-KO cells compared to the untreated (adj. *P* = 0.012), while no significant change was observed in WT cells before and after irradiation (adj. *P* = 0.37) (Supplementary Figure S4I). These results suggest that the ID3-KO cells display loss of accessibility after irradiation. An integrative analysis of our chromatin accessibility and transcriptomic data shows that genes that were upregulated in irradiated ID3-KO cells showed more accessibility at their TSSs compared to irradiated WT cells (adj. *P* *=*0.00093) (Supplementary Figure S4J). In comparison, downregulated genes displayed less accessibility compared to WT cells (adj. *P* *=*0.0056) (Supplementary Figure S4K). Next, we analyzed the chromatin accessibility at the promoters of the different DNA repair gene sets. We observed a reduction of chromatin accessibility in irradiated ID3-KO versus WT cells at promoter regions of DEGs involved in HR (adj. *P* = 0.038) and BER (adj. *P* = 0.045) pathways (Figure 5B andC), while there was no significant change for genes involved in FA (adj. *P* = 0.22), MMR (adj. *P* = 0.09), NHEJ (adj. *P* = 0.39) and NER (adj. *P* = 0.94) pathways (Supplementary Figures S4L and M, and S5A and B). Notably, this differential accessibility between ID3-KO and WT cells at the promoters of HR and BER genes was not observed before exposure to IR, but only after IR treatment. These results suggest that the loss of ID3 impairs the chromatin accessibility of gene regulatory regions of HR and BER repair genes. This change subsequently prevents the induction of those genes in response to IR.

### ID3 promotes DNA binding and transcriptional activity of E2F1, which in turn mediates the expression of HR genes in response to IR

To further elucidate potential mechanisms of gene regulation by ID3, we performed a transcription factor (TF) binding motif analysis for the differentially expressed genes in irradiated ID3-KO cells. The analysis included the promoter regions (1.5 kb up- and downstream from the transcriptional start sites (TSS)) of all downregulated genes, and identified common binding motifs for several members of the E2F TF family, in particular *E2F1*and *E2F4* (Figure 5D). We used TF data from the ENCODE Project Consortium (54) to confirm the binding of E2F1 and E2F4 at the promoter regions of all downregulated DNA repair genes (Supplementary Figure S5C). Remarkably, our transcriptome GO analysis showed downregulation ofthe E2F pathway (Figure 4B). Since the downregulated DNA repair genes exhibit common motifs for E2F1 and E2F4TFs, we analyzed the TF activity using the computational method diffTF which estimates the accessibility of TF binding sites and their putative activity. This analysis revealed reduced activity of E2F1 and E2F4, as well as other E2F members (E2F5, E2F6 and E2F8) in irradiated ID3-KO cells (Figure 5E). The reduced activity of E2F TFs is correlated with the reduced expression of their target genes in response to IR (Pearson correlation, *P* = 0.02; Figure 5F), whereas no significant correlation was observed in the untreated condition (*P* = 0.61). This suggests that the loss of E2F1 and E2F4 activity in ID3-KO cells in response to IR can reduce the induction of their target DNA repair genes. Since E2F1 is an activating transcription factor, we analyzed its enrichment at the promoters of the affected DNA repair genes *BRCA1*, *BRCA2*, *RAD51*, *RBBP8* and *EXO1*, using ChIP-qPCR. We performed four independent ChIP-qPCR experiments, two were normalized to the percentage of the input (Figure 5G) and another two were normalized to IgG enrichment (Supplementary Figure S5D). Generally following irradiation, enrichment of E2F1 was increased at the promoters of indicated genes in the WT cells, whereas in ID3-KO cells, E2F1 enrichment was not enhanced (Figure 5G). Although we could not detect a significant increase in E2F1 accumulation at the promoters of *BRCA1* and *RAD51* post IR using an IgG normalization method, we observed that ID3-KO cells display reduced E2F1 accumulation after IR (Supplementary Figure S5D).

We further asked how ID3 regulates E2F1 activity. One possibility could be *via* posttranslational modifications induced by known interaction partners like arginine methyltransferases 1 and 5 (PRMT1 and PRMT5). PRMT1 catalyzes the asymmetric methylation of E2F1 at the arginine residue R109 (55), while PRMT5 catalyzes symmetric methylation of E2F1 at two arginine residues R111 and R113 (55,56). PRMT5 is a member of the E2F pathway which was among the downregulated pathways in our transcriptome analysis in irradiated ID3-KO cells (Figure 4B). In our RNA-seq analysis, *PRMT5* expression as well as *E2F1* and its dimerization partner 1 (*TFDP1*) were downregulated in ID3-KO cells after IR. We validated the expression of *PRMT5* and *TFDP1* with RT-qPCR and Western Blot and observed that RNA and protein expression of both were induced in response to IR in the WT cells, while this induction was missing in ID3-KO cells (Figure 5H-J). Lu *et al.*, recently reported the involvement of RECQL in transcription regulation of *ESR1*, the gene encoding estrogen receptor (ERα), by enhancing the chromatin accessibility at the *ESR1* regulatory regions in cooperation with FOXA1 (57). This in turn regulates ERα-dependent gene expression. To explore whether NBS1 or RECQL are also involved in transcription regulation of DNA repair genes following IR, similar to ID3, we performed qPCR analyses for the affected HR genes after knocking down ID3, NBS1, RECQL, E2F1 and PRMT5. Depletion of either NBS1 or RECQL showed no significant effects on the expression of these HR genes, while depletion of E2F1 and PRMT5 displayed effects similar to ID3 loss (Supplementary Figure S5E–J). This indicates that ID3 promotes DNA repair via two different mechanisms: (i) it cooperates with NBS1 and RECQL to facilitate the recruitment of DNA repair factors and (ii) it works together with E2F1 and PRMT5 to regulate the expression of DNA repair genes in response to IR.

Our proteomic analysis also revealed a link between ID3 and E2F1, as the GO term ‘Transcription regulation by TP53’ was among the top GO terms. Among the players of this pathway, two additional protein interactions of ID3 were identified, namely PRMT1 and CDK2 (Supplementary Figure S5K), with whom E2F1 is interacting too (55,58). We validated the interaction of ID3 to PRMT1 (Supplementary Figure S5L). Collectively, we suggest that ID3 affects the activity of E2F1 by interaction with PRMT1/ PRMT5or *via* the CDK2 pathway and can subsequently regulate the expression of DNA repair genes in response to DNA damage.

### ID3 expression correlates with the expression score of HR-related pathways in cancer patients

**Figure 6.**
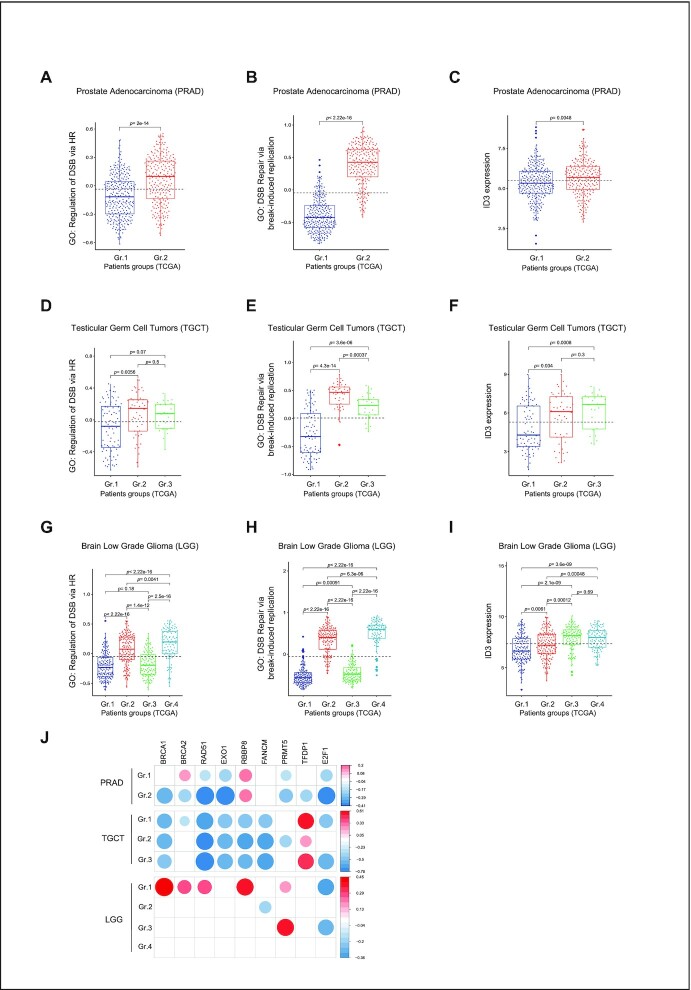
ID3 low expression is associated with impaired HR in cancer patients. (**A**) Box plot representation showing the single sample GSVA enrichment score of GO: regulation of double strand break via homologous repair in clusters of patient samples of TCGA prostate adenocarcinoma (PRAD). (**B**) Box plot representation showing the single sample GSVA enrichment score of GO: double strand break repair via break-induced replication in clusters of patient samples of TCGA PRAD. (**C**) Box plot representation showing ID3 expression as log_2_ counts per million in clusters of patient samples of TCGA PRAD. (**D**) Box plot representation showing the single sample GSVA enrichment score of GO: regulation of double strand break via homologous repair in clusters of patient samples of TCGA testicular germ cell tumors (TGCT). (**E**) Box plot representation showing the single sample GSVA enrichment score of GO: double strand break repair via break-induced replication in clusters of patient samples of TCGA TCGT. (**F**) Box plot representation showing ID3 expression as log_2_ counts per million in clusters of patient samples of TCGA TGCT. (**G**) Box plot representation showing the single sample GSVA enrichment score of GO: regulation of double strand break via homologous repair in clusters of patient samples of TCGA low grade glioma (LGG). (**H**) Box plot representation showing the single sample GSVA enrichment score of GO: double strand break repair via break-induced replication in clusters of patient samples of TCGA LGG. (**I**) Box plot representation showing ID3 expression as log_2_ counts per million in clusters of patient samples of TCGA LGG patient samples. Group comparisons were performed using an unpaired Wilcoxon rank test. The dotted lines in all panels represent the average score across all groups. All group comparisons were performed using an unpaired Wilcoxon rank test. (**J**) Bubble plot representation showing the Pearson correlation coefficient between the expression of DNA repair genes (*BRCA1*, *BRCA2*, *RAD51*, *EXO1*, *RBBP8*, *FANCM*, *PRMT5*, *TFDP1* and *E2F1*) and ID3 in clusters of TCGA patient samples of PRAD (top), TGCT (middle) and LGG (bottom). Empty squares represent no significant correlation (*P* > 0.05). The color of the circles represent the degree of correlation (red: positive; blue: negative) and the larger the size of the circle the stronger the significance.

To identify the role of low ID3 expression in tumors, we used TCGA gene expression data of cancer patients with different primary tumor entities. Single sample gene set enrichment scores were estimated for the HR-related gene ontologies DOUBLE STRAND BREAK REPAIR VIA BREAK INDUCED REPLICATION (GO:0000727), RECOMBINATIONAL REPAIR (GO:0000725), REGULATION OF DOUBLE STRAND BREAK REPAIR VIA HOMOLOGOUS RECOMBINATION (GO:0010569), and REPLICATION BORN_DOUBLE STRAND BREAK REPAIR VIA SISTER CHROMATID EXCHANGE (GO:1990414). These were further used to perform hierarchical clustering followed by dynamic tree cut to estimate the number of groups per tumor type (Supplementary Figure S6A). Our analysis identified up to five groups of patients with different gene set enrichment scores of the HR-related gene ontologies among several tumor entities (Supplementary Figure S6A). We further analyzed *ID3* expression among these groups and observed a positive association between *ID3* expression and the enrichment scores of the HR-related gene ontologies in patients of prostate adenocarcinoma (PRAD) (Figure 6A-C), testicular germ cell tumors (TGCT) (Figure 6D–F) and kidney renal papillary cell carcinoma (KIRP) (Supplementary Figure S6B–D). In low-grade glioma (LGG), thymoma (THYM), diffuse large B-cell lymphoma (DLBC) and colon adenocarcinoma (COAD), we identified groups with low *ID3* expression which also show a low enrichment score of HR-related pathways (Figure 6G–I, Supplementary Figure S6E–J and S7A–C). We further plotted the correlation between *ID3* expression in those patient groups and the expression of the key HR-related genes. A positive correlation was observed between *ID3* expression and expression of HR genes in all analyzed tumor entities (Figure 6J and Supplementary Figure S7D). These results support that low *ID3* expression is associated with impaired HR gene expression. Highlighting these results, stratification of tumor patients with aberrant *ID3* expression will allow new targeted and personalized therapeutic options to be applied.

### ID3 loss in tumor cells confers sensitivity to PARP inhibitor

**Figure 7.**
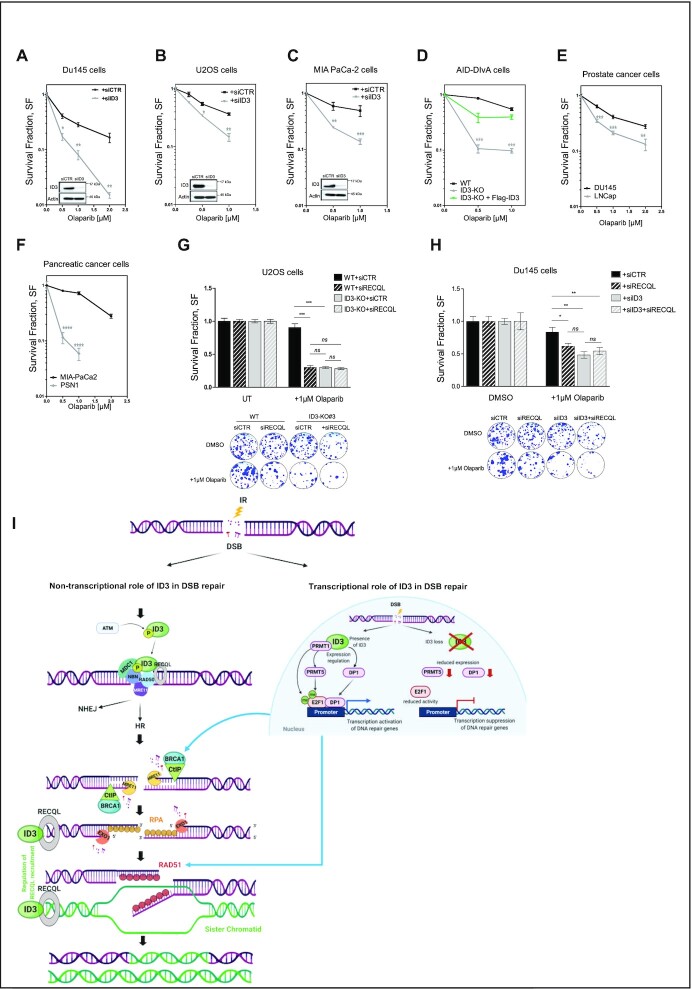
ID3 loss confers sensitivity to PARP inhibition. (**A–C**) Clonogenic survival assay of Du145, MIA-PaCa2 and U2OS cells transfected either with control siRNA (siCTR) or with a pool of four siRNAs targeting ID3 (siID3) and treated with the indicated dose of olaparib. (**D**) Clonogenic survival assay of WT and ID3-KO cells treated with the indicated doses of olaparib. (**E**, **F**) Clonogenic survival assay of various prostate cancer and pancreatic cancer cell lines expressing different ID3 levels treated with the indicated dose of olaparib. (**G**) Clonogenic survival assay of WT and ID3-KO U2OS cells transfected with siRECQL and treated with the indicated dose of olaparib, lower panel shows representative colony formation images. (**H**) Clonogenic survival assay of Du145 cells with single or double depletion of ID3 and RECQL and treated with the indicated dose of olaparib, lower panel shows representative colony formation images. All clonogenic survival assays are presented as mean ± SEM of three independent experiments, Student *t* test was performed. (**I**) Model of the multiple roles of ID3 in HR. Statistical significance is presented as: * *P* < 0.05, ** *P* < 0.01, *** *P* < 0.001, **** *P* < 0.0001, ns = not significant.

Understanding the molecular alterations of HR occurring in cancer cells has helped to achieve a more effective and durable targeted cancer therapy. An example of such targeted therapy is the application of poly-(ADP-ribose) polymerase (PARP) inhibitors for the treatment of HR repair-deficient cancers. As a proof of principle, we performed colony formation assays using cell lines with different ID3 levels to investigate whether treatment with the PARP inhibitor (PARPi) Olaparib affects cell survival. ID3 loss sensitizes the cells to PARPi (Figure 7A–D and Supplementary Figure S7E). Moreover, cell lines withlow ID3 expression were more sensitive to PARPi (Figure 7E and F). Since ID3 and RECQL cooperate to promote HR, we determined the effect of their single or simultaneous depletion on PARPi sensitivity. Consistently, single depletion of either ID3 or RECQL sensitizes the cells to PARPi, and this effect was comparable to that of their simultaneous depletion (Figure 7G and H). Thus, HR deficiency caused by ID3 loss in tumor cells confers sensitivity to PARP inhibition, which might be used in therapeutic approaches.

## DISCUSSION

A growing body of evidence suggests that ID3 is involved in the regulation of the DNA damage response (15,17,18). A previous publication from our lab (17) demonstrates a genomic instability in the form of chromosomal gain and loss in ID3-deficient tumor samples. Similarly, Lee *et al.*, reported chromosomal instability by measuring Genome-Wide DNA Copy Number Variation in ID3-Depleted MRC-5 cells (18). We employed proteomic, transcriptomic and epigenetic approaches to comprehensively study the mechanisms underlying the role of ID3 in DNA repair. In addition to the previously reported interaction of ID3 to MDC1, our proteomic analysis uncovered DNA damage-induced interactions of ID3 with proteins involved in DSB repair, namely with NBS1, RAD50 and RECQL. For the interaction with MDC1, the HLH domain of ID3 was reported to be crucial when it is phosphorylated by ATM at serine 65 (18). Our results show that the interaction of ID3 with NBS1, RAD50 and RECQL is ATM-dependent. These interactions are lost upon S65A mutation, thus underlining that the newly identified interactions of ID3 are also mediated by the ATM-dependent phosphorylation of the HLH domain of ID3. NBS1 and RAD50 are members of the MRN complex (MRE11-RAD50-NBS1) which accumulates at DSBs immediately following DNA damage, triggers DSB-sensing as well as DNA-end-tethering, and regulates the repair of DSBs by both NHEJ and HR pathways (59–66). The MRN complex interacts with ATM and MDC1 and promotes MDC1 accumulation and binding to phosphorylated γH2AX, thus magnifying DNA damage signaling around the DSB (67,68). We assume that the ATM-dependent association of ID3 with both the MRN complex and MDC1 is crucial for the initiation of DSB repair through NHEJ and HR repair, although our results show this to a different extent. Moreover, our kinetic experiments revealed that interaction of ID3 with MRN complex subunits occurred earlier than the interaction with MDC1. The depletion of MDC1 did not abolish the interaction of ID3 with RAD50, RECQL or NBS1,which may be because the MRN complex is recruited to DSBs earlier and upstream of MDC1 (62). This indicates that ID3 can bind to the MRN complex and RECQL independently of MDC1 in response to DNA damage. Whether ID3 exists in one complex with all the repair factors (NBS1, RAD50, RECQL and MDC1) or in different complexes is still unclear and needs further follow-up studies.

Similar to the loss of these crucial DNA repair factors (NBS1, RAD50 and RECQL), ID3 knockdown resulted in a high cellular sensitivity to IR, reduction of both NHEJ and HR efficiencies and higher residual γH2AX *foci*. Moreover, loss of ID3 leads to reduced chromatin binding of NBS1, RAD50 and RECQL following IR. The simultaneous knockdown of ID3 and either of these players showed a comparable high level of residual γH2AX *foci* after IR, indicating that ID3 works together with these players to promote DNA DSB repair pathways. The simultaneous depletion of ID3 and either of these players displayed, however, an additive inhibitory effect on cellular survival after IR, which can be caused by the multiple functions of these players with regard to cellular survival. DSB repair and survival after IR mostly depend on NHEJ and not HR (69). ID3 depletion had,however,a stronger impact on DSB repair after IR in S/G2 versus G1 phases of the cell cycle, i.e. on HR.

To analyze the function of ID3 in NHEJ or HR in more detail, we used the DIVA cell model. This allows the differentiated analysis of AsiSI-induced DSBs which are either prone to HR or prone to NHEJ, as DSBs close to actively transcribed genes are preferentially repaired using HR whereas breaks near inactive genes are repaired by NHEJ (19,70). We were able to show in AID-DIVA cells that ID3 enrichment was stronger at HR-prone DSBs, highlighting the function of ID3 in the repair of DSBs near actively transcribed genes. Overall, our study generated evidence to support that ID3 loss mainly affects HR. First, ID3 loss caused a dramatic reduction of the recruitment of the key HR enzymes CtIP, MRE11, pRPA (S4/S8) and RAD51 to the damaged chromatin, while NHEJ protein recruitment was only slightly reduced. Second, ID3 knockdown resulted in a high cellular sensitivity to cisplatin, an inter- and intra-strand crosslinking agent causing replication-associated DNA damage, which in turn requires the HR machinery to be adequately repaired. This is in accordance with the reported hypersensitivity of ID3-deficient colon cancer-initiating cells to the cisplatin analog oxaliplatin (16). In summary, the preference of ID3 for HR can also explain that the majority of ID3-deficient pancreatic acinar cell carcinomas were harboring mutational signatures associated with DSB repair defects, mainly resulting from HR deficiencies (17).

Mechanistically, we suggest a further downstream role of ID3 in HR. This can be explained either by the role of the MRN complex in promoting DNA end resection by MRE11 nuclease activity (71–73) or by the interaction of ID3 with RECQL. RECQL is a helicase that facilitates the recruitment of RPA to single-stranded DNA at stalled replication forks. Its catalytic activity is associated with DSB repair *via* HR to maintain genome integrity. RECQL possesses DNA branch migration activity to resolve aberrant recombination intermediates and D-loops to facilitate completion of HR (74–80). In line with these data, we demonstrate that RECQL is required for efficient HR. Dependent on ID3, it accumulates at HR-prone DSBs generated by AsiSI restriction enzyme, and it exerts its helicase functions to facilitate chromatin unwinding to promote end resection and RAD51 loading. Both proteins act together in HR *via* promoting RAD51 loading, as double depletion of both enzymes displayed similar reduction of IR-induced RAD51 *foci* to their single knockdown in S/G2 cells. In contrast to the effects of double depletion of ID3 and NBS1, single depletion of RAD50 or MDC1 indicated that both players promote RAD51 loading in different ways than ID3 and RECQL.

Our proteomic analysis further showed an interaction of ID3 with several factors involved in transcription initiation and elongation, which is consistent with the known involvement of ID3 in the regulation of gene transcription. We therefore interrogated whether ID3 regulates the expression of DNA repair genes. A recent study reports an integrative analysis of driver gene mutations and gene expression profiles of Burkitt lymphomas (BL) (81) and the authors identified ID3 as one of the BL driver genes. Silencing mutations in ID3 were associated with low enrichment scores of DNA repair pathway gene sets. Western blot analysis by Lee *et al.* showed, however, no effect of ID3 knockdown on the expression of several DNA repair proteins in different cell lines (18). Upon comparing RNA-seq data of ID3-KO and WT cells, we could not identify expression changes in DNA repair genes when the cells were untreated. Upon IR exposure, however, we identified downregulated DNA repair genes in the ID3-KO cells. Several DNA repair genes were consistently shown to be induced following IR (82–86). This induction was also observed in our WT cells after IR but it was impaired in irradiated ID3-depleted cells. Noteworthy, the majority of those genes are active in HR and FA pathways.

Our results suggest at least two possible explanations of why the expression of those DNA repair genes is not induced in cells lacking ID3. First, the reduced chromatin accessibility of their promoters in ID3-KO cells could impede expression induction after exposure to IR. This agrees with our proteomic analysis showing the interaction of ID3 with chromatin remodeling factors (Figure 1B and Supplementary Tables S2 and S3). These results are in line with a recent study that performed ATAC-seq in ID3-null mice and showed a repressive effect of ID3 loss on the chromatin accessibility. The authors attributed their observation to a possible interaction of ID3 with various specific TFs (87). How this interaction exactly mediates the chromatin accessibility in response to DNA damage requires further investigation. Second, the activity and expression of the crucial activating transcription factor E2F1 were altered in ID3-KO cells and correlated with the expression of its target genes only in response to IR. E2F1 is known to regulate genes involved in DNA repair, cell cycle and apoptosis (88–93). E2F1 alterations can subsequently impair the transient induction of the expression of HR and FA genes in response to IR. Our ChIP-qPCR analysis following IR demonstrates that ID3-depleted cells exhibit reduced DNA binding of the transcription factor E2F1 at the promoters of the HR genes *BRCA1*, *BRCA2*, *RAD51*, *RBBP8* and *EXO1*. In the WT cells, we observed enhanced binding of E2F1 at 30 minutes after IR at the promoters of *BRCA2*, *RBBP8* and *EXO1*, but not at the promoters of *BRCA1* and *RAD51*; however, at the *BRCA1* promoter we observed an increase in E2F1 enrichment after 1h following IR. This suggests a contribution of an additional regulatory factor on the top of E2F1 to regulate the induction of DNA repair gene expression following IR. Possible candidates can be derived from data on the regulation of E2F1 activity that has been intensively studied. Thus, it is well established that active E2F1 forms a heterodimer with DP1. The DNA-binding domain of DP1 facilitates the sequence-specific DNA binding of E2F1 (94,95). In addition, PRMT5 is known to methylate the arginine residues R111/R113 in E2F1 which regulates E2F1’s transcriptional activity (55,56,96). Inactivation of PRMT5 altered E2F1 activity, resulting in downregulation of E2F1 target genes, such as cell cycle and DNA repair genes (96). Following IR, we show here that ID3 depletion reduces the expression of the regulators of E2F1 activity, DP1 and PRMT5, which results in an impaired E2F1 transcriptional activity and, consequently, downregulation of DNA repair genes. This is in line with a recent report showing that depletion of PRMT5 impairs the expression of DNA repair genes following IR (86). Apart from its role in regulating E2F1, PRMT5 has emerging epigenetic functions in the control of DNA repair. It can contribute to DNA repair either by binding to the proximal promoter regions of DNA repair genes and promoting their transcription, which then may explain our E2F1 ChIP-qPCR results at the promoters of *BRCA1* and *RAD51* (86), or by regulating the splicing of DNA repair genes and key histone-modifying enzymes such as TIP60 (97,98). To identify the link between ID3 and the PRMT5-E2F1 pathway, we scrutinized our proteomic analysis data revealing two protein interactions to ID3 following IR, namely PRMT1 and CDK2, with whom E2F1 is interacting too (55,58). Altogether, our experimental results suggest an epigenetic role of ID3 by which it contributes to the regulation of the chromatin environment in response to DNA damage. This could include both a direct or indirect interaction of ID3 with epigenetic enzymes such as PRMT1/PRMT5; however, the precise molecular mechanisms on how ID3 and PRMT1/PRMT5 function together to control the induction of DNA repair gene expression requires further investigation.

To identify the impact of low ID3 expression on DSB repair particularly by HR in tumors, we used TCGA gene expression data of various tumor entities. Three tumor types, namely PRAD, TGCT and KIRP, showed direct correlation between ID3 expression and the enrichment score of HR-related pathways. In patients with LGG, Thym, DLBC and COAD, we also found at least one subgroup of patients with low ID3 expression and low HR enrichment score, indicating that low ID3 expression in specific subgroups of cancer patients is associated with impaired HR-related pathways. Such subgroups with low HR might specifically be targeted by therapeutic approaches. One example of such approach is the selective targeting of HR-deficient cancer cells by PARP inhibitors (PARPi), which represents the so-called synthetic lethality concept (99–101). Cells harboring one of the two gene or protein defects are viable while cells containing both defects are nonviable. According to our findings that cells lacking ID3 lose the ability to localize RAD51 to DSBs and are unable to perform HR, we show that PARPi treatment selectively targets cells with low or absent ID3 expression. Mechanistically, in the absence of ID3, PARPi-induced replication-associated DSBs will not be repaired via the error-free HR. These breaks will instead be inaccurately repaired through NHEJ or left unrepaired, thus leading to cell death. This offers new therapeutic options for the treatment of cancers lacking ID3 expression. A future comprehensive identification and stratification of tumor entities characterized by aberrant ID3 expression will be necessary to enable anticancer clinical trials with PARP-inhibitors (102–104). Our results show that ID3 and RECQL are acting together in HR and promote RAD51 loading in both U2OS and Du145 cells. Consistently, the colony formation assay data demonstrate that RECQL knockdown resulted in PARPi sensitivity in these cell lines, although to a different extent. Simultaneous knockdown of ID3 and RECQL shows similar effects as the single knockdown of ID3. This suggests that ID3 and RECQL are cooperating in HR. RECQL is additionally required to cope with replication stress, Berti *et al.* reported that after TOP1 inhibition RECQ1 is promoting replication fork restart, and RECQL-depleted cells showed sensitivity to Camptothecin and etoposide (105). The RECQL– PARP1 axis is critical for both replication restart and DNA repair. During replication stress activation of PARP1 stabilizes the forks in the reversed state by transient inhibition of RECQL-dependent fork restart until the damage is repaired (106). Sharma *et al.* showed that depletion of RECQL leads to modest HR deficiency and mild PARPi sensitivity (75) when cell survival is measured 72 h after PARPi treatment. In contrast, Viziteu *et al.* reported that RECQL depletion clearly sensitizes multiple myeloma cells to PARPi (107). As both studies used similar cell growth assays, these data suggest that sensitivity to PARPi in absence of RECQL may be cell type-specific and it has to be carefully investigated in the future using alternative methods.

In summary, we conclude that ID3 has a multifaceted regulatory function in DNA repair, in particular in HR. This includes association with DSB repair core players such as the MRN complex and MDC1 that facilitates the early events of the DNA damage response and initiation of end resection, a prerequisite step for DSB repair via HR. Moreover, ID3 interacts and cooperates with RECQL to promote further downstream steps of HR (Figure 7I). In addition, ID3 exhibits a transcriptional regulatory role mediated by the transcription factors E2F1 to promote the expression of DNA repair genes in response to ionizing radiation. Highlighting the identified mechanisms, ID3 loss confers cellular sensitivity to PARPi and offers a promising approach for the treatment of tumors harboring ID3 alterations.

## DATA AVAILABILITY

The data that support the findings of this study are presented in the main text paper and in the online Supplementary Information file. Further information and requests for resources and reagents should be directed to and will be fulfilled by the corresponding authors, Ali Bakr (a.bakr@dkfz.de). Most of the analyses are based on publicly available software listed in the respective methods section. The mass spectrometry proteomics data have been deposited to the ProteomeXchange Consortium via the PRIDE [1] partner repository with the dataset identifier PXD028946. The source codes of custom scripts were deposited at following repository: https://github.com/HeyLifeHD/ID3_DNArepair. The RNA-seq and ATAC-seq datasets generated during this study are available at European Genome-Phenome Archive (EGA) with the accession number EGAS00001004478.

## Supplementary Material

gkab964_Supplemental_Files
