# Correction to ‘ID3 promotes homologous recombination via non-transcriptional and transcriptional mechanisms and its loss confers sensitivity to PARP inhibition’

**DOI:** 10.1093/nar/gkad877

**Published:** 2023-10-04

*Nucleic Acids Research*, Volume 49, Issue 20, 18 November 2021, Pages 11666–11689, https://doi.org/10.1093/nar/gkab964

In the originally published version of this manuscript, the highlighted micrograph was mistakenly used in Figure 2M:


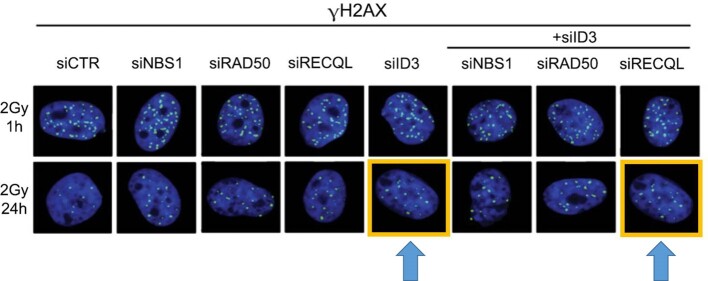


This has now been corrected in a new Figure 2M, which is provided below. This correction does not affect the results nor the drawn conclusions.


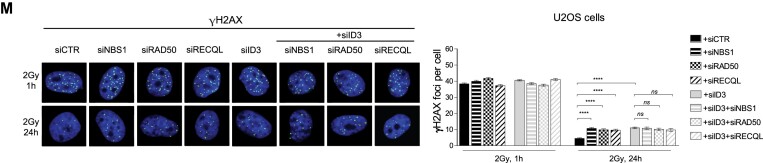


These details have been corrected only in this correction notice to preserve the published version of record.

