# Supplementary material for: ID3 promotes homologous recombination via non-transcriptional and transcriptional mechanisms and its loss confers sensitivity to PARP inhibition
Source: Nucleic Acids Res. 2021 Oct 28;49(20):11666–89. doi: 10.1093/nar/gkab964 (PMC8599806; doi:10.1093/nar/gkab964)
Supplement: gkab964_Supplemental_Files [file gkab964_supplemental_files.zip › gkab964_Supplementary_information_and_figures.pdf]

Supplemental Tables Titles

Table S1: list of the identified proteins in the untreated samples.

Table S2: list of the identified proteins in the irradiated samples which were collected 15 minutes.

Table S3: list of the uniquely identified proteins in the irradiated samples which were collected 1h post irradiation.

Table S4: dataset of the Venn diagram.

Table S5: list of enriched proteins_IR_15min vs UT

Table S6: list of enriched proteins_IR_1h vs UT

Table S7: list identified DNA repair proteins

Table S8: sequences of oligonucleotides such as small interfering RNA (siRNAs) and primers.

Table S9: data sets of the differentially expressed genes (DEGs) in the untreated ID3-KO cells.

Table S10: data sets of the differentially expressed genes (DEGs) in the irradiated ID3-KO cells.

Supplemental Figures Titles and Legends

Figure S1: (A) Schematic diagram showing the binding positions of two CRISPR gRNAs targeting ID3 Exon 1 in AID-DIvA and U2OS cells, sequencing results displaying the gene editing at the binding sites of the gRNAs, and Western blot analysis of different ID3-KO clones and re-introduction of GFP-Flag-HA-tagged ID3 in ID3-KO cells. **(B)** Western blot showing the distribution of Flag-tagged ID3 in cytoplasmic and nuclear fractions after irradiation. Experiments were repeated three times. **(C)** Western blot to confirm the immunoprecipitation of Flag-tagged ID3 using Flag-beads. Experiments were done three times. **(D)** Western blot showing the distribution of endogenous ID3 in cytoplasmic and nuclear fractions after irradiation. **(E)** Western blot showing the inhibition of phosphorylation of ATM upon treatment with ATM inhibitor. **(F)** Pathway enrichment analysis using all interaction proteins identified in untreated (IP-ID3 vs IP-empty vector). Analysis was done using Metascape resources (Ref.# 71). **(G)** Pathway enrichment analysis using the interaction proteins identified in IR_15min (IP-ID3 vs IP-empty vector). Analysis was done using Metascape resources (Ref.# 71).

Figure S2: (A) Pathway enrichment analysis using the interaction proteins identified in IR_1h (IP-ID3 vs IP-empty vector). Analysis was done using Metascape resources (Ref.# 71). **(B)** Volcano plots showing all identified ID3 interaction candidates, identified proteins are highlighted in red. Y-axis represents *t*-test statistics of log-10 LFQ intensities. **(C)** Rank plots showing the enrichment of the proteins compared to the respective control IP. Proteins were ranked from higher to lower ratios. **(D)** Western blot of co-IP of endogenous ID3 showing no interaction with CtIP or RAD51. **(E)** Clonogenic survival assay of WT and ID3-KO cells treated with the indicated dose of ionizing radiation. n=3 independent experiments; data are presented as mean ±SEM, One-Way ANOVA with Bonferroni's multiple comparison test. **(F)** Quantification of γH2AX foci in WT and different ID3-KO cells. n=3 independent experiments; data are presented as mean ±SEM, One-Way ANOVA with Bonferroni's multiple comparison test. **(G)** Quantification of γH2AX foci in WT, ID3-KO and ID3-rescue cells. n=3 independent experiments; data are presented as mean ±SEM, One-Way ANOVA with Bonferroni's multiple comparison test. **(H)** Clonogenic survival assay of pancreatic cancer cell lines with different ID3 expression levels treated with the indicated doses of ionizing radiation. n=3 independent experiments; data are presented as mean ±SEM, One-Way ANOVA with Bonferroni's multiple comparison test. **(I)** Quantification of IR-induced γH2AX foci in pancreatic cancer cell lines with different ID3 expression levels treated with 2Gy, ca. 500 cells were counted at the indicated

time points. n=3 independent experiments; data are presented as mean ±SEM, One-Way ANOVA with Bonferroni's multiple comparison test. **(J)** Representative micrographs showing ID3 staining before and after IR treatment in U2OS cells. **(K)** Enrichment of ID3 and γH2AX at ISce-I-induced DSBs in U2OS-DR cells, measured by ChIP-qPCR. Cells were treated either with DMSO or 10µM ATMi. n=3 independent experiments; data are presented as mean ±SEM, Student *t* test was used. **(L)** Western blot showing single and double knockdown efficiencies of the indicated proteins in U2OS-EJ5 and U2OS-DR cells. Experiment was independently repeated. **(M)** Western blot showing single and double knockdown efficiencies of the indicated proteins in U2OS cells. Experiment was independently repeated. **(N)** Quantification of IR-induced γH2AX foci in U2OS cells transfected with the indicated siRNAs and treated with 2Gy, ca. 500 cells were counted at the indicated time points. n=3 independent experiments; data are presented as mean ±SEM, One-Way ANOVA with Bonferroni's multiple comparison test. Statistical significance is presented as: ** = p<0.05, ** = p<0.01, *** = p<0.001, **** = p<0.0001*, *ns= not significant*.

Figure S3: **(A)** Enrichment of ID3 at individual NHEJ-prone and HR-prone DSBs in AID-DIvA cells measured by ChIP-qPCR. n=3 independent experiments; data are presented as mean ±SD. **(B)** Western blot demonstrating the expression of RPA at the indicated time points after IR in U2OS cells. Experiment was independently repeated. **(C)** Western blot representing the expression of several DNA repair proteins in WT and ID3-depleted U2OS cells. Experiment was independently repeated. **(D)** Western blot showing the knockdown efficiencies of ID3 in Du145 and U2OS cells. Experiment was independently repeated. **(E)** Quantification of IR-induced RAD51 foci in MIA-PaCa-2, Du145, LNCap and PSN-1 cells, ca. 500 cells were counted at the indicated time points. n=3 independent experiments; data are presented as mean ±SEM, One-Way ANOVA with Bonferroni's multiple comparison test. **(F)** Bar plot showing cell cycle analysis in Du145 and U2OS cells after 2Gy. **(G)** HR efficiency measured in U2OS-DR cells using the indicated siRNAs. n=3 independent experiments; data are presented as mean ±SD, Student's *t* test is performed to compare single knockdown of ID3 and the double knockdowns. **(H & I)** Representative micrographs and quantification of IR-induced RAD51 foci in Du145 and U2OS cells, respectively. ca. 500 cells were counted at indicated time points. n=3 independent experiments; data are presented as mean ±SEM, Student's *t* test is performed to compare single knockdown of ID3 and the double knockdowns. **(J)** Enrichment of RECQL at HR-prone DSBs in WT and ID3-KO AID-DIVA cells, measured by ChIP-qPCR. n=3 independent experiments; data are presented as mean ±SEM, One-Way ANOVA with Bonferroni's multiple comparison test. Statistical significance is presented as: ** = p<0.05, ** = p<0.01, *** = p<0.001, **** = p<0.0001, ns= not significant*.

Figure S4: **(A)** Barcode plot showing gene set enrichment analyses of DNA repair pathways in untreated ID3-KO cells compared to untreated WT cells. NES= Normalized enrichment score. **(B)** Heat map showing the differentially expressed DNA repair genes in the untreated condition. **(C)** Gene Ontology enrichment analysis of the significantly upregulated genes in irradiated ID3-KO cells. **(D)** Heat map showing the differentially expressed genes in untreated WT, ID3-KO and MDC1 knockdown cells. **(E)** Upset plot showing the different expression pattern between ID3-depleted and MDC1-depleted cells. **(F & G)** RT-qPCR expression analyses of *BRCA2* and *FANCM* genes in WT and ID3-KO cells with or without IR treatment (5Gy) at the indicated time points; error bars represent the mean ±SEM, One-Way ANOVA with Dunnett's multiple comparison test to compare all to the WT untreated. **(H)** Scatter plot showing the correlation between differential chromatin accessibility at promoter regions and gene expression in untreated cells. **(I)** Mean of the chromatin accessibility at all transcription start sites (TSSs) of protein coding genes measured by ATAC-seq. n=3 for WT and KO cells. **(J & K)** Mean chromatin accessibility at TSSs of downregulated and upregulated genes in irradiated ID3-KO cells measured by ATAC-seq (n=3). **(L & M)** Mean chromatin accessibility at promoter regions of genes involved in FA and MMR, respectively, measured by ATAC-seq (n=3). Statistical significance is presented as: ** = p<0.05, ** = p<0.01, *** = p<0.001, **** = p<0.0001, ns= not significant*.

Figure S5: **(A & B)** Mean chromatin accessibility at promoter regions of genes involved in NER and NHEJ, respectively, measured by ATAC-seq (n=3). **(C)** UCSC genome browser screen shots of the promoter regions of several DNA repair genes showing common binding sites of E2F1 and E2F4. **(D)** Enrichment of E2F1 at the promoters of the indicated genes in WT and ID3-KO cells, measured by ChIP-qPCR. n=2 independent experiments; data are presented as mean of 6 technical replicates from two

independents experiments ±SEM, Student's *t* test. **(E-I)** RT-qPCR expression analysis of *BRCA1, BRCA2, RAD51, RBBP8 (CtIP),* and *EXO1* genes in U2OS cells with IR treatment (5Gy) upon knockdown of indicated genes, n=5, data presented as mean ±SD, Student's *t* test to compare the knockdown effect to the corresponding siCTR. **(J)** RT-qPCR expression analysis of *NBN, RECQL, E2F1* and *PRMT5* genes to confirm the knockdown efficiency in U2OS cells with IR treatment (5Gy), n=5, data presented as mean ±SD, Student's *t* test to compare the knockdown effect to the corresponding siCTR. **(K)** Volcano plots showing identified ID3 interaction candidates, highlighting PRMT1. Y-axis represents t-test statistics of log-10 LFQ intensities. **(L)** Western blot to confirm co-IP of endogenous ID3 to PRMT1. Statistical significance is presented as: ** = p<0.05, ** = p<0.01, *** = p<0.001, **** = p<0.0001, ns= not significant*.

Figure S6: (A) Heatmap representation of the single sample GSVA enrichment scores of gene ontologies representing regulation of double strand breaks via homologous repair, recombinational repair, double strand break repair via break-induced replication and replication-born double strand break repair via sister chromatid exchange in clusters of TCGA patient samples of PRAD, TGCT and LGG, kidney renal papillary carcinoma (KIRP), Thymoma (THYM), Diffuse Large B-Cell Lymphoma (DLBC) and Colon Adenocarcinoma (COAD). **(B)** Box plot representation showing the single sample GSVA enrichment score of GO: regulation of double strand break via homologous repair in clusters of patient samples of TCGA KIRP. **(C)** Box plot representation showing the single sample GSVA enrichment score of GO: double strand break repair via break-induced replication in clusters of patient samples of TCGA KIRP. **(D)** Box plot representation showing ID3 expression as log2 counts per million in clusters of patient samples of TCGA KIRP. **(E)** Box plot representation showing the single sample GSVA enrichment score of GO: regulation of double strand break via homologous repair in clusters of patient samples of TCGA THYM. **(F)** Box plot representation showing the single sample GSVA enrichment score of GO: double strand break repair via break-induced replication in clusters of patient samples of TCGA THYM. **(G)** Box plot representation showing ID3 expression as log2 counts per million in clusters of patient samples of TCGA THYM. **(H)** Box plot representation showing the single sample GSVA enrichment score of GO: regulation of double strand break via homologous repair in clusters of patient samples of TCGA DLBC. Group comparisons were performed using an unpaired Wilcoxon rank test. The dotted line represents the average score across all groups. **(I)** Box plot representation showing the single sample GSVA enrichment score of GO: double strand break repair via break-induced replication in clusters of patient samples of TCGA DLBC. **(J)** Box plot representation showing ID3 expression as log2 counts per million in clusters of patient samples of TCGA DLBC. All the dotted lines represents the average score across all groups.All group comparisons were performed using an unpaired Wilcoxon rank test.

Figure S7: (A) Box plot representation showing the single sample GSVA enrichment score of GO: regulation of double strand break via homologous repair in clusters of patient samples of TCGA COAD. **(B)** Box plot representation showing the single sample GSVA enrichment score of GO: double strand break repair via break-induced replication in clusters of patient samples of TCGA COAD. **(C)** Box plot representation showing ID3 expression as log2 counts per million in clusters of patient samples of TCGA COAD. **(D)** Bubble plot representation showing the Pearson correlation coefficient between the expression of DNA repair genes (BRCA1, BRCA2, RAD51, EXO1, RBBP8, FANCM, PRMT5, TFDP1 and E2F1) and ID3 in clusters of TCGA patient samples of KIRP, THYM, DLBC and COAD. Empty squares represent no significant correlation ($p > 0.05$). The color of the circles represent the degree of correlation (red: positive; blue: negative) and the larger the size of the circle the stronger the significance. All the dotted lines represents the average score across all groups.All group comparisons were performed using an unpaired Wilcoxon rank test. **(E)** Representative images of clonogenic survival assay of indicated cells transfected either with control siRNA (siCTR) or with a pool of 4 siRNAs targeting ID3 (siID3) and treated with the indicated dose of olaparib. **(F)** Western blot showing the efficiencies of single and double depletion of ID3 and RECQL in U2OS and Du145 cells. Experiment was independently repeated.

Figure S1

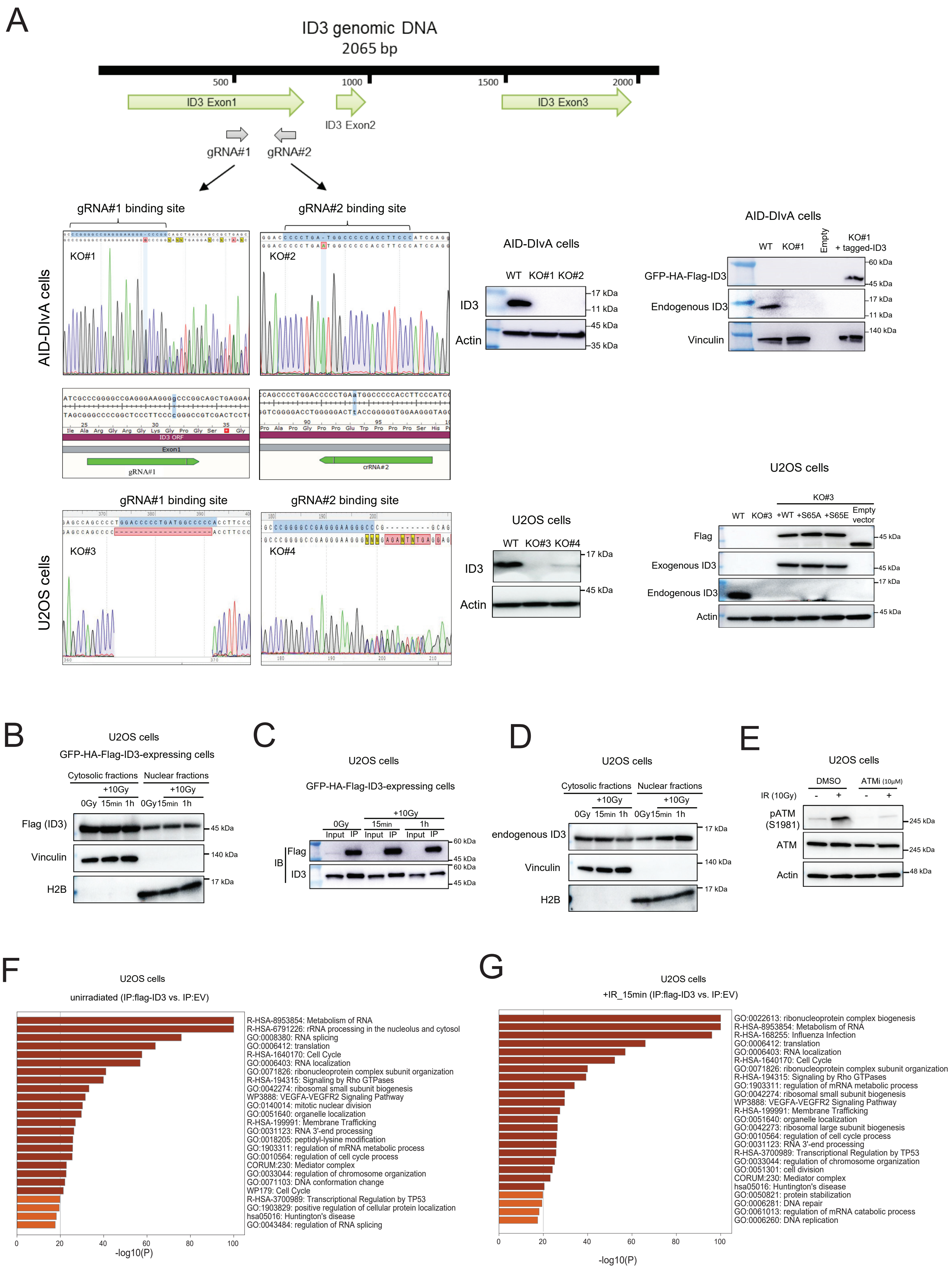
A
ID3 genomic DNA
2065 bp
500
1000
1500
2000
ID3 Exon1
ID3 Exon2
ID3 Exon3
gRNA#1
gRNA#2
gRNA#1 binding site
gRNA#2 binding site
AID-DIvA cells
KO#1
KO#2
gRNA#1
crRNA#2
Exon1
ID3 ORF
U2OS cells
KO#3
KO#4
AID-DIvA cells
WT KO#1 KO#2
ID3
Actin
17 kDa
11 kDa
45 kDa
35 kDa
AID-DIvA cells
WT KO#1 Empty KO#1 + tagged-ID3
GFP-HA-Flag-ID3
Endogenous ID3
Vinculin
60 kDa
45 kDa
17 kDa
11 kDa
140 kDa
U2OS cells
WT KO#3 KO#4
ID3
Actin
17 kDa
45 kDa
U2OS cells
KO#3
WT KO#3 +WT +S65A +S65E Empty vector
Flag
Exogenous ID3
Endogenous ID3
Actin
45 kDa
17 kDa
B
U2OS cells
GFP-HA-Flag-ID3-expressing cells
Cytosolic fractions
Nuclear fractions
+10Gy
0Gy 15min 1h
Flag (ID3)
Vinculin
H2B
45 kDa
140 kDa
17 kDa
C
U2OS cells
GFP-HA-Flag-ID3-expressing cells
+10Gy
0Gy 15min 1h
Input IP
IB
Flag
ID3
60 kDa
45 kDa
D
U2OS cells
Cytosolic fractions
Nuclear fractions
+10Gy
0Gy 15min 1h
endogenous ID3
Vinculin
H2B
17 kDa
140 kDa
E
U2OS cells
DMSO
ATMi (10μM)
IR (10Gy)
pATM (S1981)
ATM
Actin
245 kDa
48 kDa
F
U2OS cells
unirradiated (IP:flag-ID3 vs. IP:EV)
R-HSA-8953854: Metabolism of RNA
R-HSA-6791226: rRNA processing in the nucleolus and cytosol
GO:0008380: RNA splicing
GO:0006412: translation
R-HSA-1640170: Cell Cycle
GO:0006403: RNA localization
GO:0071826: ribonucleoprotein complex subunit organization
R-HSA-194315: Signaling by Rho GTPases
GO:0042274: ribosomal small subunit biogenesis
WP3888: VEGFA-VEGFR2 Signaling Pathway
GO:0140014: mitotic nuclear division
GO:0051640: organelle localization
R-HSA-199991: Membrane Trafficking
GO:0031123: RNA 3'-end processing
GO:0018205: peptidyl-lysine modification
GO:1903311: regulation of mRNA metabolic process
GO:0010564: regulation of cell cycle process
CORUM:230: Mediator complex
GO:0033044: regulation of chromosome organization
GO:0071103: DNA conformation change
WP179: Cell Cycle
R-HSA-3700989: Transcriptional Regulation by TP53
GO:1903829: positive regulation of cellular protein localization
hsa05016: Huntington's disease
GO:0043484: regulation of RNA splicing
0 20 40 60 80 100
-log10(P)
G
U2OS cells
+IR_15min (IP:flag-ID3 vs. IP:EV)
GO:0022613: ribonucleoprotein complex biogenesis
R-HSA-8953854: Metabolism of RNA
R-HSA-168255: Influenza Infection
GO:0006412: translation
GO:0006403: RNA localization
R-HSA-1640170: Cell Cycle
GO:0071826: ribonucleoprotein complex subunit organization
R-HSA-194315: Signaling by Rho GTPases
GO:1903311: regulation of mRNA metabolic process
GO:0042274: ribosomal small subunit biogenesis
WP3888: VEGFA-VEGFR2 Signaling Pathway
R-HSA-199991: Membrane Trafficking
GO:0051640: organelle localization
GO:0042273: ribosomal large subunit biogenesis
GO:0010564: regulation of cell cycle process
GO:0031123: RNA 3'-end processing
R-HSA-3700989: Transcriptional Regulation by TP53
GO:0033044: regulation of chromosome organization
GO:0051301: cell division
CORUM:230: Mediator complex
hsa05016: Huntington's disease
GO:0050821: protein stabilization
GO:0006281: DNA repair
GO:0061013: regulation of mRNA catabolic process
GO:0006260: DNA replication
0 20 40 60 80 100
-log10(P)

Figure S2

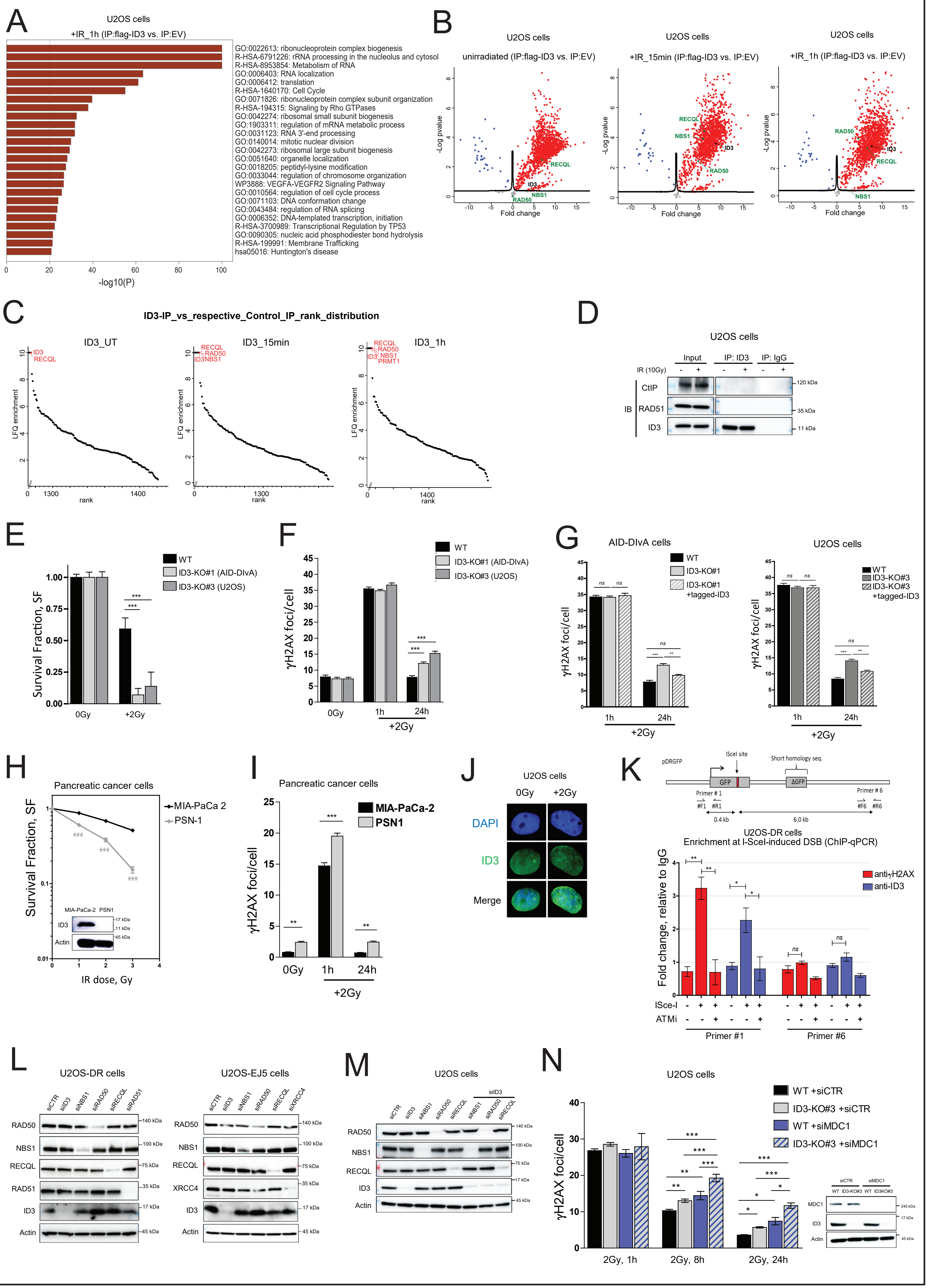

A
U2OS cells
+IR_1h (IP:flag-ID3 vs. IP:EV)
GO:0022613: ribonucleoprotein complex biogenesis
R-HSA-6791226: rRNA processing in the nucleolus and cytosol
R-HSA-8953854: Metabolism of RNA
GO:0006403: RNA localization
GO:0006412: translation
R-HSA-1640170: Cell Cycle
GO:0071826: ribonucleoprotein complex subunit organization
R-HSA-194315: Signaling by Rho GTPases
GO:0042274: ribosomal small subunit biogenesis
GO:1903311: regulation of mRNA metabolic process
GO:0031123: RNA 3'-end processing
GO:0140014: mitotic nuclear division
GO:0042273: ribosomal large subunit biogenesis
GO:0051640: organelle localization
GO:0018205: peptidyl-lysine modification
GO:0033044: regulation of chromosome organization
WP3888: VEGFA-VEGFR2 Signaling Pathway
GO:0010564: regulation of cell cycle process
GO:0071103: DNA conformation change
GO:0043484: regulation of RNA splicing
GO:0006352: DNA-templated transcription, initiation
R-HSA-3700989: Transcriptional Regulation by TP53
GO:0090305: nucleic acid phosphodiester bond hydrolysis
R-HSA-199991: Membrane Trafficking
hsa05016: Huntington's disease
-log10(P)
B
U2OS cells
unirradiated (IP:flag-ID3 vs. IP:EV)
+IR_15min (IP:flag-ID3 vs. IP:EV)
+IR_1h (IP:flag-ID3 vs. IP:EV)
-Log pvalue
Fold change
RECQL
NBS1
RAD50
ID3
C
ID3-IP_vs_respective_Control_IP_rank_distribution
ID3_UT
ID3_15min
ID3_1h
LFQ enrichment
rank
PRMT1
D
U2OS cells
Input
IP: ID3
IP: IgG
IR (10Gy)
IB
CtIP
RAD51
ID3
120 kDa
35 kDa
11 kDa
E
Survival Fraction, SF
WT
ID3-KO#1 (AID-DIvA)
ID3-KO#3 (U2OS)
0Gy
+2Gy
F
γH2AX foci/cell
1h
24h
G
AID-DIvA cells
U2OS cells
ID3-KO#1 +tagged-ID3
ID3-KO#3 +tagged-ID3
H
Pancreatic cancer cells
MIA-PaCa 2
PSN-1
MIA-PaCa-2
PSN1
Actin
17 kDa
11 kDa
45 kDa
IR dose, Gy
I
Pancreatic cancer cells
MIA-PaCa-2
PSN1
J
U2OS cells
DAPI
ID3
Merge
K
pDRGFP
IScel site
Short homology seq.
GFP
ΔGFP
Primer # 1
Primer # 6
#F1
#R1
#F6
#R6
0.4 kb
6.0 kb
U2OS-DR cells
Enrichment at I-SceI-induced DSB (ChIP-qPCR)
Fold change, relative to IgG
anti-γH2AX
anti-ID3
ISce-I
ATMi
Primer #1
Primer #6
L
U2OS-DR cells
U2OS-EJ5 cells
siCTR
siID3
siNBS1
siRAD50
siRECQL
siRAD51
siXRCC4
RAD50
NBS1
RECQL
RAD51
XRCC4
ID3
Actin
140 kDa
100 kDa
75 kDa
35 kDa
17 kDa
45 kDa
M
U2OS cells
siID3
N
U2OS cells
WT +siCTR
ID3-KO#3 +siCTR
WT +siMDC1
ID3-KO#3 +siMDC1
2Gy, 1h
2Gy, 8h
2Gy, 24h
MDC1
245 kDa

Figure S3

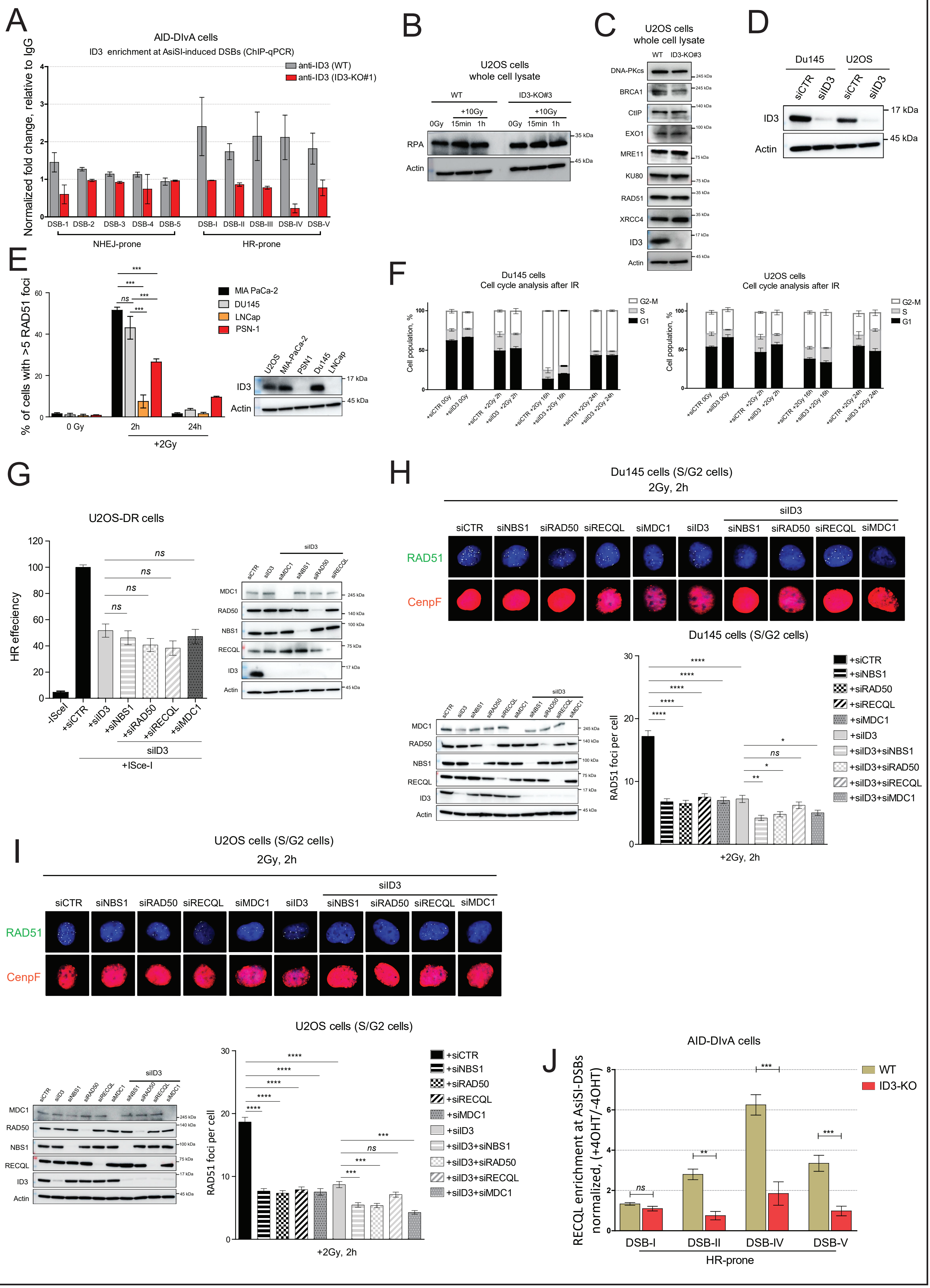

A
AID-DIvA cells
ID3 enrichment at AsiSI-induced DSBs (ChIP-qPCR)
anti-ID3 (WT)
anti-ID3 (ID3-KO#1)
Normalized fold change, relative to IgG
DSB-1 DSB-2 DSB-3 DSB-4 DSB-5 DSB-I DSB-II DSB-III DSB-IV DSB-V
NHEJ-prone
HR-prone
B
U2OS cells
whole cell lysate
WT
ID3-KO#3
+10Gy
0Gy 15min 1h
RPA
Actin
C
U2OS cells
whole cell lysate
DNA-PKcs
BRCA1
CtIP
EXO1
MRE11
KU80
RAD51
XRCC4
ID3
Actin
D
Du145
U2OS
siCTR
siID3
ID3
Actin
17 kDa
45 kDa
E
% of cells with >5 RAD51 foci
MIA PaCa-2
DU145
LNCap
PSN-1
0 Gy
2h
24h
+2Gy
F
Du145 cells
Cell cycle analysis after IR
U2OS cells
Cell cycle analysis after IR
Cell population, %
G2-M
S
G1
G
U2OS-DR cells
HR effeciency
-ISceI
+siCTR
+siID3
+siNBS1
+siRAD50
+siRECQL
+siMDC1
siID3
+ISce-I
H
Du145 cells (S/G2 cells)
2Gy, 2h
siCTR siNBS1 siRAD50 siRECQL siMDC1 siID3 siNBS1 siRAD50 siRECQL siMDC1
RAD51
CenpF
Du145 cells (S/G2 cells)
RAD51 foci per cell
+siCTR
+siNBS1
+siRAD50
+siRECQL
+siMDC1
+siID3
+siID3+siNBS1
+siID3+siRAD50
+siID3+siRECQL
+siID3+siMDC1
+2Gy, 2h
I
U2OS cells (S/G2 cells)
2Gy, 2h
U2OS cells (S/G2 cells)
J
AID-DIvA cells
RECQL enrichment at AsiSI-DSBs
normalized, (+4OHT/-4OHT)
WT
ID3-KO
DSB-I DSB-II DSB-IV DSB-V
HR-prone

Figure S4

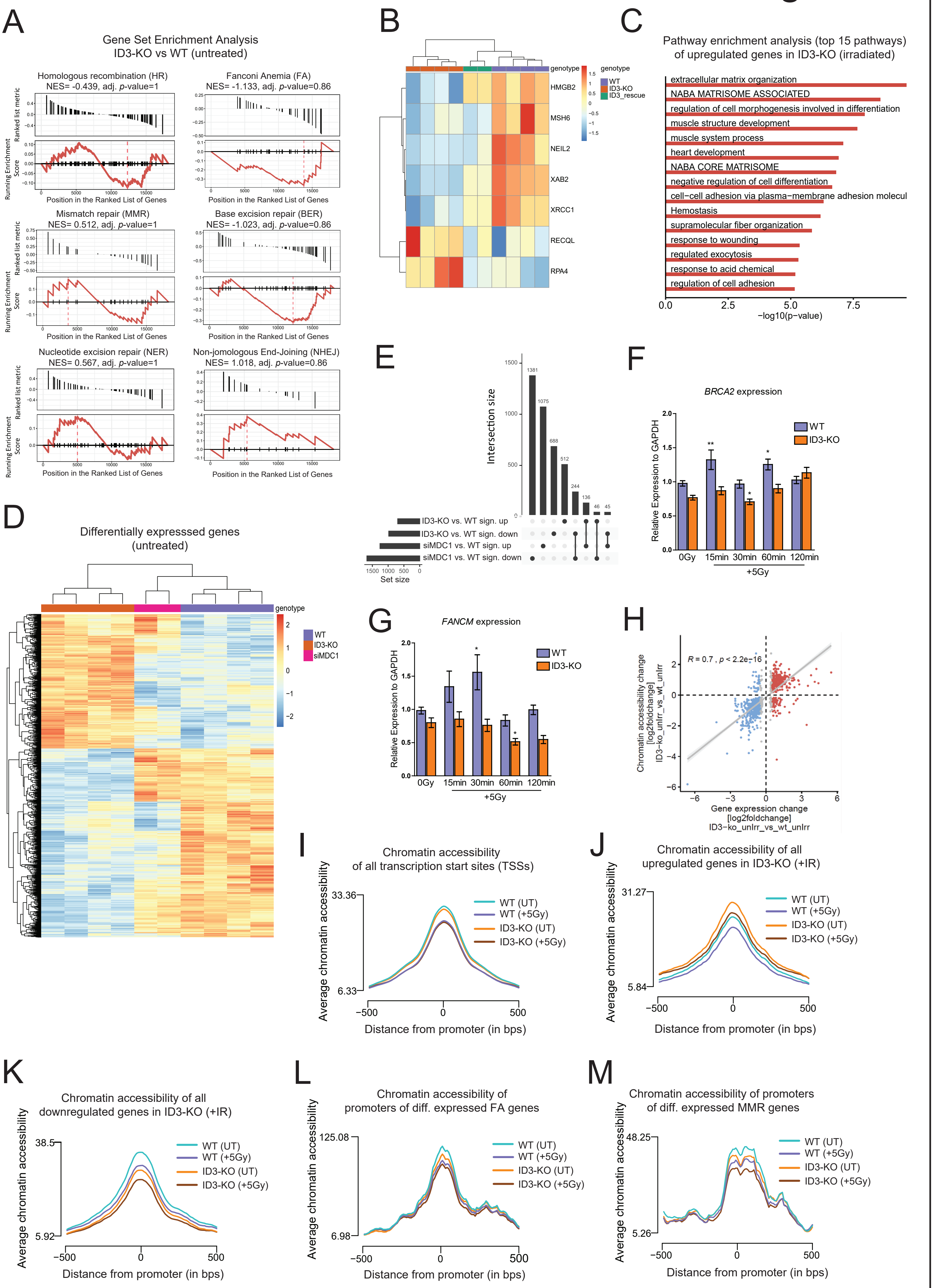

A
Gene Set Enrichment Analysis
ID3-KO vs WT (untreated)
Homologous recombination (HR)
NES= -0.439, adj. p-value=1
Fanconi Anemia (FA)
NES= -1.133, adj. p-value=0.86
Mismatch repair (MMR)
NES= 0.512, adj. p-value=1
Base excision repair (BER)
NES= -1.023, adj. p-value=0.86
Nucleotide excision repair (NER)
NES= 0.567, adj. p-value=1
Non-jomologous End-Joining (NHEJ)
NES= 1.018, adj. p-value=0.86
Ranked list metric
Running Enrichment Score
Position in the Ranked List of Genes
B
genotype
WT
ID3-KO
ID3_rescue
HMGB2
MSH6
NEIL2
XAB2
XRCC1
RECQL
RPA4
C
Pathway enrichment analysis (top 15 pathways)
of upregulated genes in ID3-KO (irradiated)
extracellular matrix organization
NABA MATRISOME ASSOCIATED
regulation of cell morphogenesis involved in differentiation
muscle structure development
muscle system process
heart development
NABA CORE MATRISOME
negative regulation of cell differentiation
cell−cell adhesion via plasma−membrane adhesion molecul
Hemostasis
supramolecular fiber organization
response to wounding
regulated exocytosis
response to acid chemical
regulation of cell adhesion
−log10(p−value)
D
Differentially expresssed genes
(untreated)
genotype
WT
ID3-KO
siMDC1
E
Intersection size
1381
1075
688
512
244
136
46
45
ID3-KO vs. WT sign. up
ID3-KO vs. WT sign. down
siMDC1 vs. WT sign. up
siMDC1 vs. WT sign. down
Set size
F
BRCA2 expression
Relative Expression to GAPDH
WT
ID3-KO
0Gy
15min
30min
60min
120min
+5Gy
G
FANCM expression
Relative Expression to GAPDH
WT
ID3-KO
0Gy
15min
30min
60min
120min
+5Gy
H
R = 0.7 , p < 2.2e−16
Chromatin accessibility change
[log2foldchange]
ID3−ko_unIrr_vs_wt_unIrr
Gene expression change
[log2foldchange]
ID3−ko_unIrr_vs_wt_unIrr
I
Chromatin accessibility
of all transcription start sites (TSSs)
Average chromatin accessibility
Distance from promoter (in bps)
WT (UT)
WT (+5Gy)
ID3-KO (UT)
ID3-KO (+5Gy)
J
Chromatin accessibility of all
upregulated genes in ID3-KO (+IR)
K
Chromatin accessibility of all
downregulated genes in ID3-KO (+IR)
L
Chromatin accessibility of
promoters of diff. expressed FA genes
M
Chromatin accessibility of promoters
of diff. expressed MMR genes

Figure S5

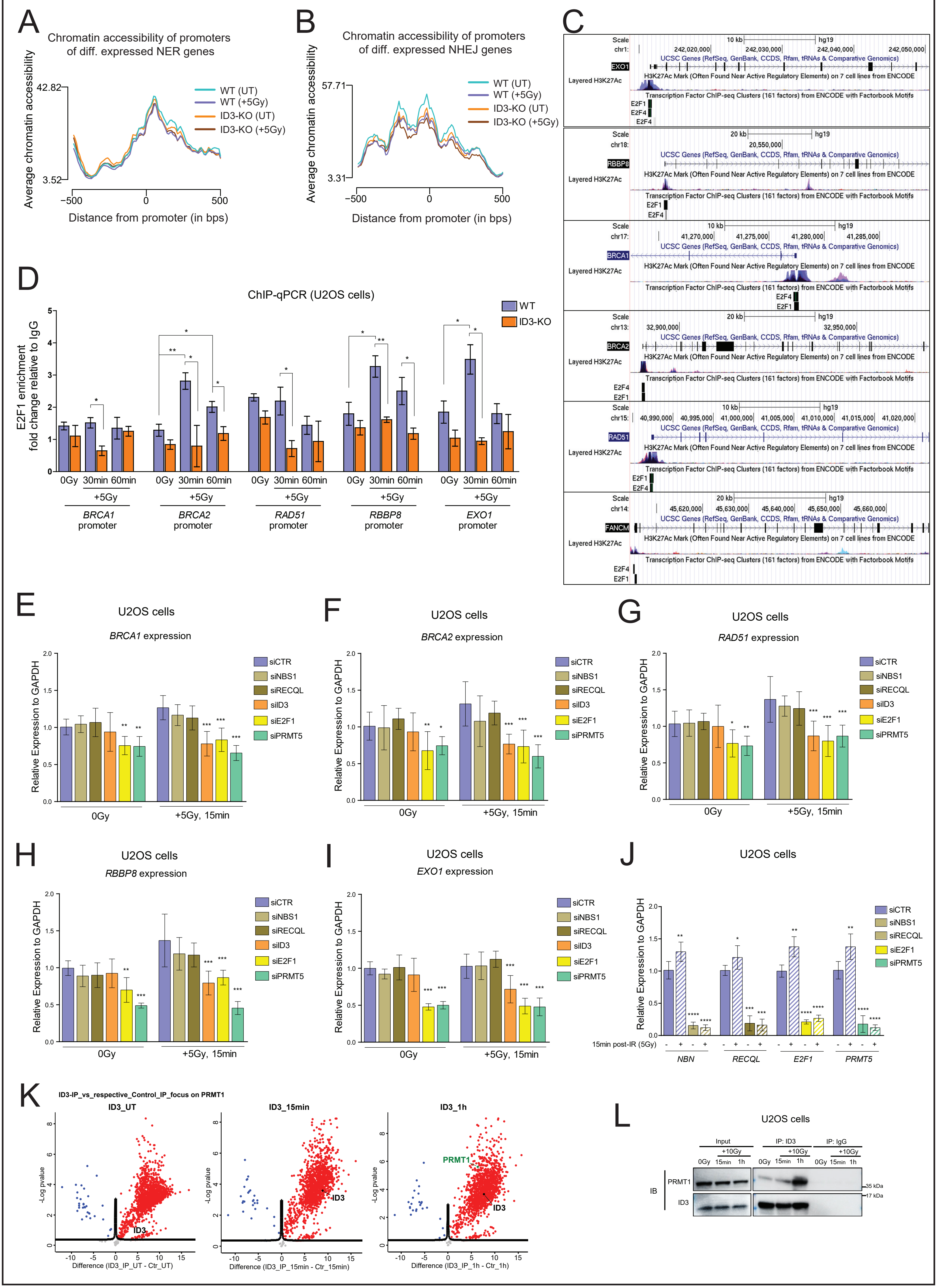
A
Chromatin accessibility of promoters of diff. expressed NER genes
Average chromatin accessibility
Distance from promoter (in bps)
WT (UT)
WT (+5Gy)
ID3-KO (UT)
ID3-KO (+5Gy)
B
Chromatin accessibility of promoters of diff. expressed NHEJ genes
C
EXO1
RBBP8
BRCA1
BRCA2
RAD51
FANCM
Layered H3K27Ac
UCSC Genes (RefSeq, GenBank, CCDS, Rfam, tRNAs & Comparative Genomics)
Transcription Factor ChIP-seq Clusters (161 factors) from ENCODE with Factorbook Motifs
E2F1
E2F4
D
ChIP-qPCR (U2OS cells)
E2F1 enrichment fold change relative to IgG
WT
ID3-KO
0Gy 30min 60min
+5Gy
BRCA1 promoter
BRCA2 promoter
RAD51 promoter
RBBP8 promoter
EXO1 promoter
E
U2OS cells
BRCA1 expression
Relative Expression to GAPDH
siCTR
siNBS1
siRECQL
siID3
siE2F1
siPRMT5
0Gy
+5Gy, 15min
F
BRCA2 expression
G
RAD51 expression
H
RBBP8 expression
I
EXO1 expression
J
15min post-IR (5Gy)
NBN
RECQL
E2F1
PRMT5
K
ID3-IP_vs_respective_Control_IP_focus on PRMT1
ID3_UT
ID3_15min
ID3_1h
-Log pvalue
Difference (ID3_IP_UT - Ctr_UT)
Difference (ID3_IP_15min - Ctr_15min)
Difference (ID3_IP_1h - Ctr_1h)
PRMT1
ID3
L
U2OS cells
Input
IP: ID3
IP: IgG
+10Gy
0Gy 15min 1h
IB
PRMT1
ID3
35 kDa
17 kDa

Figure S6

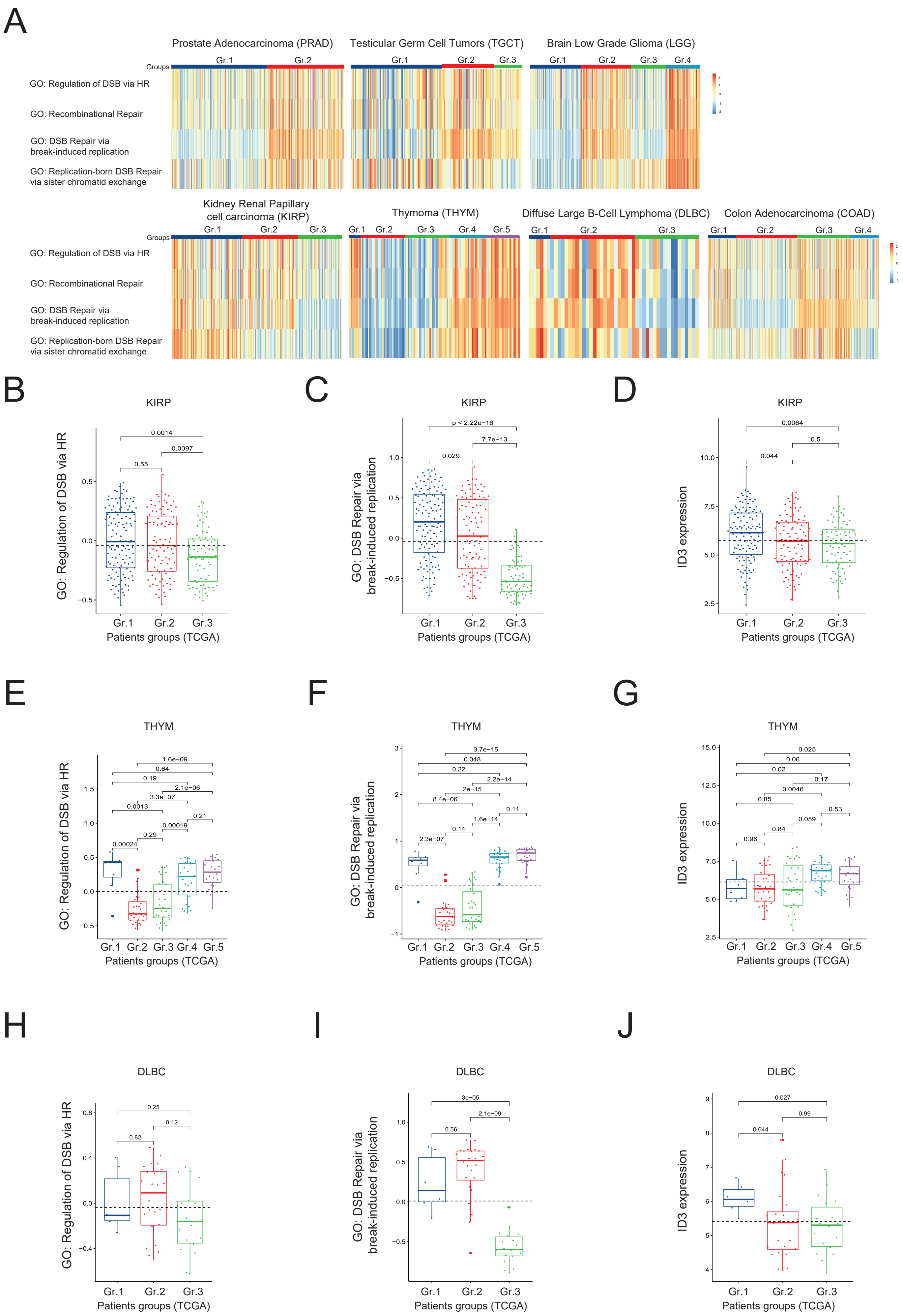

A
Prostate Adenocarcinoma (PRAD)
Testicular Germ Cell Tumors (TGCT)
Brain Low Grade Glioma (LGG)
Groups
GO: Regulation of DSB via HR
GO: Recombinational Repair
GO: DSB Repair via break-induced replication
GO: Replication-born DSB Repair via sister chromatid exchange
Kidney Renal Papillary cell carcinoma (KIRP)
Thymoma (THYM)
Diffuse Large B-Cell Lymphoma (DLBC)
Colon Adenocarcinoma (COAD)
B
KIRP
GO: Regulation of DSB via HR
Patients groups (TCGA)
C
KIRP
GO: DSB Repair via break-induced replication
Patients groups (TCGA)
D
KIRP
ID3 expression
Patients groups (TCGA)
E
THYM
GO: Regulation of DSB via HR
Patients groups (TCGA)
F
THYM
GO: DSB Repair via break-induced replication
Patients groups (TCGA)
G
THYM
ID3 expression
Patients groups (TCGA)
H
DLBC
GO: Regulation of DSB via HR
Patients groups (TCGA)
I
DLBC
GO: DSB Repair via break-induced replication
Patients groups (TCGA)
J
DLBC
ID3 expression
Patients groups (TCGA)

Figure S7

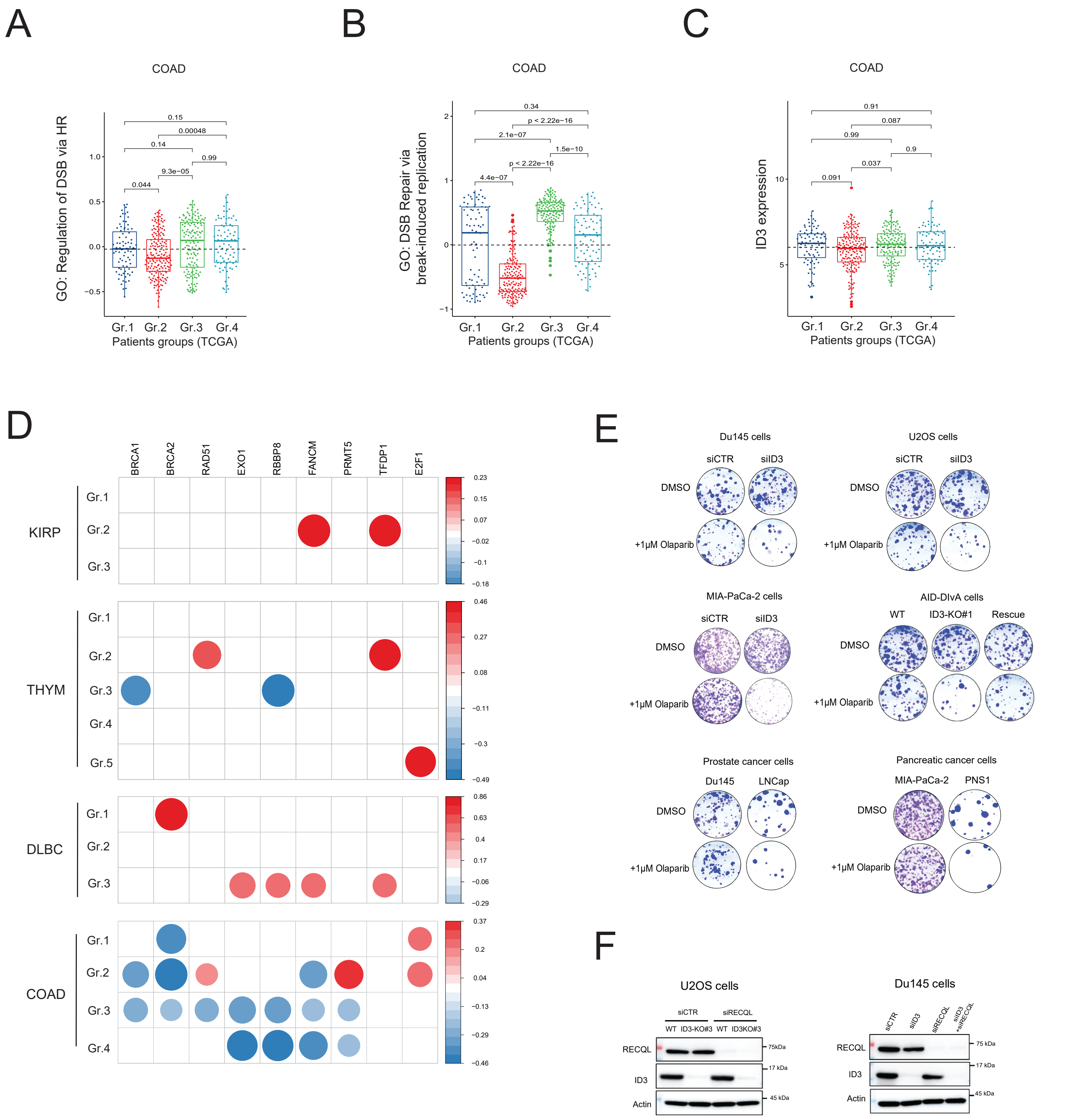

A
COAD
GO: Regulation of DSB via HR
Gr.1 Gr.2 Gr.3 Gr.4
Patients groups (TCGA)
B
COAD
GO: DSB Repair via break-induced replication
Patients groups (TCGA)
C
COAD
ID3 expression
Patients groups (TCGA)
D
BRCA1 BRCA2 RAD51 EXO1 RBBP8 FANCM PRMT5 TFDP1 E2F1
KIRP
THYM
DLBC
COAD
E
Du145 cells
U2OS cells
siCTR siID3
DMSO
+1μM Olaparib
MIA-PaCa-2 cells
AID-DIvA cells
WT ID3-KO#1 Rescue
Prostate cancer cells
Du145 LNCap
Pancreatic cancer cells
MIA-PaCa-2 PNS1
F
U2OS cells
siCTR siRECQL
WT ID3-KO#3 WT ID3KO#3
RECQL
ID3
Actin
Du145 cells
siCTR siID3 siRECQL siID3 +siRECQL
